# Feasibility study of early outpatient review and early cardiac rehabilitation after cardiac surgery: mixed-methods research design—a study protocol

**DOI:** 10.1136/bmjopen-2019-035787

**Published:** 2019-12-29

**Authors:** Dumbor Ngaage, Natasha Mitchell, Alexandra Dean, Claire Hirst, Enoch Akowuah, Patrick Joseph Doherty, Caroline Fairhurst, Kate Flemming, Catherine Hewitt, Sebastian Hinde, Alex Mitchell, Simon Nichols, Judith Watson

**Affiliations:** 1 Castle Hill Hospital, Hull University Teaching Hospitals NHS Trust, Hull, UK; 2 York Trials Unit, Department of Health Sciences, University of York, York, UK; 3 James Cook Hospital, South Tees Hospitals NHS Foundation Trust, Middlesbrough, UK; 4 Department of Health Sciences, University of York, York, UK; 5 Centre for Health Economics, University of York, York, UK; 6 Centre for Sport and Exercise Science, Sheffield Hallam University - Collegiate Crescent Campus, Sheffield, UK

**Keywords:** rehabilitation medicine, cardiothoracic surgery, statistics & research methods, health economics

## Abstract

**Introduction:**

Following cardiac surgery, patients currently attend an outpatient review 6 weeks after hospital discharge, where recovery is assessed and suitability to commence cardiac rehabilitation (CR) is determined. CR is then started from 8 weeks. Following a median sternotomy, cardiac surgery patients are required to refrain from upper body exercises, lifting of heavy objects and other strenuous activities for 12 weeks. A delay in starting CR can prolong the recovery process, increase dependence on family/carers and can cause frustration. However, current guidelines for activity and exercise after median sternotomy have been described as restrictive, anecdotal and increasingly at odds with modern clinical guidance for CR. This study aims to examine the feasibility of bringing forward outpatient review and starting CR earlier.

**Methods and analyses:**

This is a multicentre, randomised controlled, open feasibility trial comparing postoperative outpatient review 6 weeks after hospital discharge, followed by CR commencement from 8 weeks (control arm) versus, postoperative outpatient review 3 weeks after hospital discharge, followed by commencement of CR from 4 weeks (intervention arm). The study aims to recruit 100 eligible patients, aged 18–80 years who have undergone elective or urgent cardiac surgery involving a full median sternotomy, over a 7-month period across two centres. Feasibility will be measured by consent, recruitment, retention rates and attendance at appointments and CR sessions. Qualitative interviews with trial participants and staff will explore issues around study processes and acceptability of the intervention and the findings integrated with the feasibility trial outcomes to inform the design of a future full-scale randomised controlled trial.

**Ethics and dissemination:**

Ethics approval was granted by East Midlands—Derby Research Ethics Committee on 10 January 2019. The findings will be presented at relevant conferences disseminated via peer-reviewed research publications, and to relevant stakeholders.

**Trial registration number:**

ISRCTN80441309

**Strengths and limitations of this study**

- This is one of the first studies to look at the timing of follow-up and cardiac rehabilitation after cardiac surgery.
- This feasibility study is a small multicentre randomised controlled trial (RCT).
- The study will collect both qualitative and quantitative data.
- If it is determined that a larger scale RCT is feasible, this study will generate valuable data to enable its design.
- This feasibility study is not large enough to determine effectiveness or cost-effectiveness and is limited to assessing the feasibility of a larger trial.

## Introduction

Following cardiac surgery, patients currently attend their first outpatient review 6 weeks after hospital discharge, where recovery is assessed and ability to commence cardiac rehabilitation (CR) is determined. CR is then started from 8 weeks. In a 2017 survey, 35 of the 42 UK cardiac centres who responded, confirmed this as current practice.^1^ This interval before review and CR extends the period of inactivity of patients, and medical attention for surgery-related complications is often sought during this period.^2–4^ Our prospective observational study (FORCAST6) found that 39% of patients reported surgery-related complications in the 6 weeks following discharge, with the majority occurring in the first 4 weeks; 15% required hospital readmission. Although the majority of patients were satisfied with the 6 weeks interval, 44% felt it was too long and would have liked an earlier review.^1^

The standard access for heart operations is through a median sternotomy.^5–8^ In the UK, 35 158 heart operations were performed in 2015.^9^ Following a median sternotomy, patients are required to refrain from upper body exercises, lifting of heavy objects and other strenuous activities for 12 weeks.^10–13^ These ‘sternal precautions’ (SP) are intended to aid healing of the sternum. CR, which has significant short-term and long-term benefits after cardiac surgery,^14 15^ is therefore delayed. The delay can mitigate the benefits of CR,^16^ contributing to physical deconditioning, and hinder the ability of CR to facilitate timely recovery of fitness and physical activity status.^17^ However, current guidelines for activity and exercise after median sternotomy have been described as restrictive, conflicting, sometimes arbitrary, frequently anecdotal and frequently at odds with modern clinical guidance.^11^

Following surgery, there are variations in types of exercises permitted, limits for weight of objects that can be lifted, guidelines for activities and timeline for resumption of driving. Parker *et al*^18^ demonstrated that the force elicited on the breastbone by coughing far exceeded lifting above the recommended limit. Adams *et al*^11^ investigated forces associated with 32 activities of daily living and reported that the majority not restricted by SP, such as opening and closing doors, generated forces greater than the allowed weight limit. While SP may help to support bone healing, the optimal nature and duration are unclear especially since sternal bone healing occurs by 5 weeks.^12^ According to the 2013 UK national audit of CR, late commencement contributes to a substantial number of heart surgery patients declining to participate.^19^ Adverse outcomes following heart operations and other forms of surgery have been shown to be reduced by early patient review after hospital discharge.^20 21^ The FORCAST6 study showed a high incidence of postoperative complications with the current 6-week patient review after heart surgery. This was the highest in the first week after hospital discharge and declined to lowest levels by 4 weeks.^1^ It, therefore, seems logical to conduct patient reviews after hospital discharge sooner than current practice to enable early CR from the period of postoperative stability and sternal bone healing at 5 weeks after surgery (4 weeks after hospital discharge).

The British Association for Cardiovascular Prevention and Rehabilitation (BACPR) Standards and Core Components^22^ and the National Certification Programme for CR^23^ recommend early commencement of CR. This is monitored by the National Audit of CR 2018.^19^ A delay in starting CR can prolong the recovery process, increase dependence on family/carers and can cause frustration. This may contribute to anxiety and depression that is reported in patients recovering after cardiac surgery.^24^

This study aims to examine the feasibility of bringing forward outpatient review and CR in order to facilitate recovery, physical fitness and quality of life.

## Methods and analysis

### Study design

A multicentre, randomised controlled, open feasibility trial using mixed methods to establish the feasibility of conducting a study where:

- Participants have a postoperative outpatient review 6 weeks after hospital discharge, followed by commencement of CR from 8 weeks (control arm) or
- Participants have a postoperative outpatient review 3 weeks after hospital discharge, followed by commencement of CR from 4 weeks (intervention arm).

**Figure 1:**
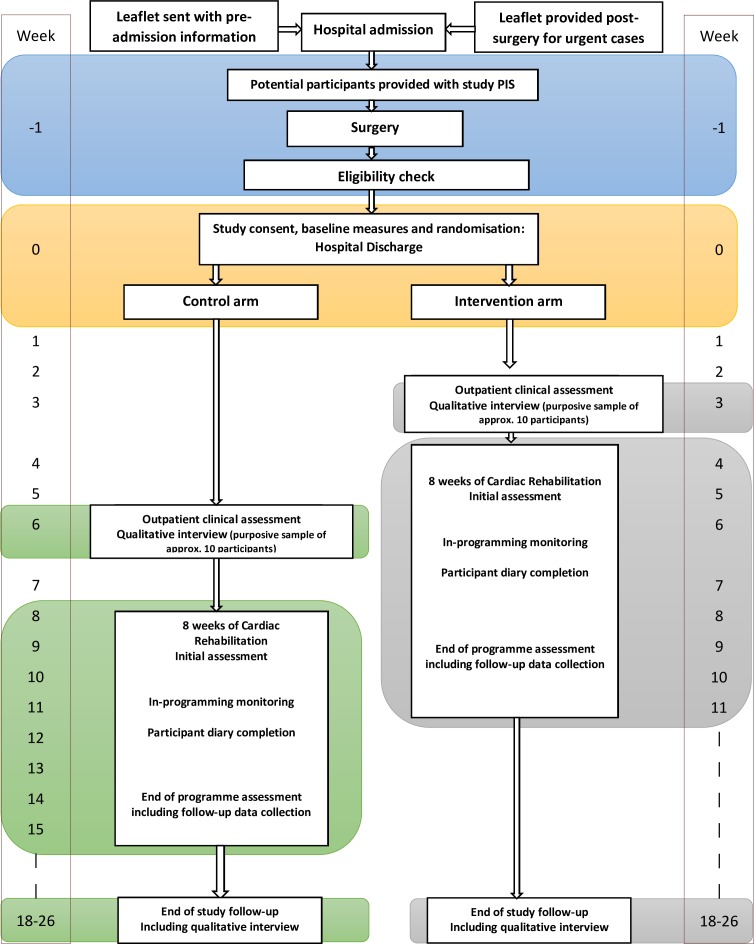
FARSTER study flow chart. PIS, Patient Information Sheet.

The study is detailed in figure 1: study flow chart and the protocol follows the Standard Protocol Items: Recommendations for Interventional Trials reporting guidelines.^25^

### Study aim

To establish the feasibility of delivering and evaluating a definitive randomised controlled trial (RCT) of the current 8th vs 4th week CR commencement pathways, reducing the period over which patients are required to refrain from usual physical activities following cardiac surgery.

### Study objectives

The primary objective is to determine the feasibility of delivering outpatient review 3 weeks after discharge postcardiac surgery, followed by CR from 4 weeks.

#### Secondary objectives

- Assess surgeons’ and surgical practitioners’ willingness to conduct outpatient review 3 weeks after discharge and refer patients to CR.
- Examine barriers to patient enrolment.
- Identify recruitment rates and drop-out to follow-up.
- Identify the most appropriate outcome measures.
- Test follow-up procedures and data collection tools and management.
- Assess the feasibility of conducting an economic evaluation of any future definitive RCT.
- Gather outcome data for power and sample size calculations for the RCT.
- Inform any necessary redesign of a new recovery pathway in light of information gained.

### Study population and setting

Patients attending for cardiac surgery at Hull University Teaching Hospitals National Health Service (NHS) Trust, UK and South Tees Hospitals NHS Foundation Trust, UK will be approached regarding study participation.

### Inclusion criteria

- Undergone elective or urgent cardiac surgery, including the following procedures (isolated or combined):
  - Coronary artery bypass graft (CABG).
  - Aortic valve replacement.
  - Mitral valve repair or replacement.
  - Atrial fibrillation ablation.
  - Left atrial appendage occlusion.
- Has had a full median sternotomy.
- Willing and capable of giving informed consent.
- Aged 18–80 years at the time of giving consent.
- Able to self-complete the English language outcome measure tools (or can complete with assistance).
- Able to follow detailed verbal instructions required for clinical assessments.

### Exclusion criteria

- Body mass index (BMI) >45 kg/m^2^.
- Heart failure with left ventricular ejection fraction of <30% before surgery.
- Early postoperative sternal wound complications (eg, deep sternal wound infection, wound dehiscence and/or sternal instability).
- Postoperative complications resulting in prolonged hospital stay >14 days after surgery.

### Amendment to eligibility criteria

During the initial recruitment period, fewer patients than anticipated were meeting the eligibility criteria, and many were ‘out of area’ impacting on their ability to attend study CR locations. With the funder’s approval, a substantial amendment was submitted to the ethics committee. The original criteria had only included patients who had undergone CABG, had an upper age limit of 75 years and a maximum BMI limit of 40 kg/m^2^. This amendment was approved on 30 October 2019.

### Sample size

This study is a feasibility RCT and therefore does not have a primary outcome measure to inform a power calculation. Sample sizes of between 24 and 70 have been recommended for feasibility trials to allow for the reliable estimation of an SD for use in future sample size calculations.^26 27^ The plan is to recruit 100 eligible patients, allowing for a 30% attrition rate in order to still have 70 patients in the final analysis. There will be a 7-month recruitment period across two centres.

### Participant recruitment

Patients scheduled for elective cardiac surgery will be given the study leaflet prior to surgery. Patients undergoing urgent cardiac surgery will receive it following surgery. All patients will undergo surgery as normal, receiving standard in-hospital postoperative care.

### Eligibility and consent

After discharge from intensive care to the ward following surgery, the medical staff will review patients’ routine clinical examination results, including sternal stability and postoperative tests. Those identified as potentially eligible, interested and willing, will have their eligibility confirmed by the research nurse using an eligibility checklist and written informed consent will be obtained according to Good Clinical Practice guidelines. Consent will also be sought to contact participants who are interested in being interviewed as part of an embedded qualitative study.

Patients screened as eligible, but who develop complications between being considered ready for discharge and going home, will not proceed to consent until deemed well enough (if remain in hospital for >14 days after surgery, as per exclusion criteria they will not proceed at all).

### Randomisation

Following surgery, consent and completion of baseline data collection and assessments, participants will be randomised to either the control or intervention arm.

Randomisation will be performed by a remote, centralised randomisation service provided by York Trials Unit (YTU), and the allocation sequence generated by a statistician not involved in the study. Participants will be individually randomised and stratified according to study site on a 1:1 basis using variable block sizes. Authorised site research staff will telephone the randomisation service to obtain the participant’s allocation.

### Outcomes

Participant self-reported data will be collected at baseline, prior to CR commencement, following CR completion and at final follow-up assessment (up to 26 weeks postrandomisation):

1. Demographics (baseline only).
2. EQ-5D-5L (EuroQol-5 dimensions-5 levels): a validated generic patient-reported outcome measure.^28^
3. NHS resource use.

The following clinical data will be collected:

1. Height, weight, BMI, preoperative presentation (baseline only).
2. Heart rate, blood pressure, oxygen saturation at baseline, outpatient review and pre-CR as per usual practice.
3. Physical fitness assessed by dynamic testing with incremental shuttle walk test (ISWT) for both study arms at commencement and end of CR, and at final follow-up.
4. Cardiopulmonary fitness assessed by cardiopulmonary exercise testing (CPET) at baseline and at final follow-up, for up to 25 patients in each study arm (Hull study site only).
5. 30 and 90-day mortality, surgical site complications and hospital readmission rates.

In line with the objectives of a feasibility study, we will also gather information on:

1. Recruitment rates and drop-out to follow-up.
2. Compliance to treatment arm allocation.
3. Acceptability of patient recruitment, early outpatient review and CR to patients, clinicians and NHS organisations.

### Cardiopulmonary exercise testing

Maximal CPET provides a holistic assessment of the cardiovascular, ventilatory and metabolic responses to exercise and is a powerful diagnostic and prognostic tool.^29^ Maximal CPET is the gold-standard method of assessing changes in aerobic fitness, which is potentially important if we are to assess the beneficial/not harmful effects of early CR. Maximal CPET will be conducted on a cycle ergometer with patients pedalling at 50 revolutions per minute throughout the test. The test workload will start at 0watts and increase in 10watts/minute increments until the patient reaches volitional exhaustion (ideally 8–12 min^29^). Maximal CPET will only be carried out (after randomisation) in up to 25 participants in each arm in the Hull site only due to costs and logistics, but also to ensure that test procedures and data interpretation are consistent. CPET will be completed once a participant is deemed clinically stable and conducted within 14 days after surgery. It will be repeated at the final follow-up.

### Postoperative outpatient review

As standard practice, all participants will have a postoperative outpatient clinical review in order to be certified suitable to commence CR. This clinical decision is usually based on the absence of limiting complications. This review will take place at 6 weeks posthospital discharge in the control arm, and 3 weeks postdischarge in the intervention arm. Participants considered unfit for CR will be given a second review appointment for approximately 1 week later and if necessary, a third review appointment 2 weeks later. Reason(s) they were deemed unfit will be documented. If they are deemed unfit for CR at the third review, they will not commence CR as part of the Feasibility Study of Early Outpatient Review and Early Cardiac Rehabilitation After Cardiac Surgery: Mixed Methods Research Design (FARSTER) study. They will, however, continue to be monitored as per usual practice and be sent all study questionnaires for completion.

### Pre-CR assessment

Referred participants will be offered a comprehensive programme and an assessment prior to starting their programme. Participants will receive a holistic assessment from a specialist physiotherapist or exercise professional including exercise testing using the ISWT to help personalise their exercise prescription. The ISWT is a popular aerobic fitness assessment tool in UK CR programmes and recommended by the CR physiotherapist association.^30 31^ Although reference values for ISWT are available in conventional CR patient populations,^32^ they have not yet been validated for use in the early CR cohorts. If a participant misses this appointment, they will be sent another.

### CR programme

This will consist of supervised low-to-moderate intensity exercise performed once (South Tees) or twice (Hull) a week for 8 weeks (as is standard UK practice). Exercise will be prescribed according to standards published by BACPR.^22^ Attendance at these sessions and session content will be recorded for each participant.

### Post-CR appointment

Following completion of the CR programme (16 weeks postdischarge for the control arm and 12 weeks postdischarge for the intervention arm), all participants will have a repeat ISWT. If a participant misses this appointment, they will be sent another. A discharge letter will be sent to participants’ general practitioners summarising their treatment.

### Final follow-up assessment

At final follow-up (up to 26 weeks after randomisation), participants will have a repeat CPET (if appropriate), undergo a final ISWT, have an end-of-study consultation and be examined for sternal wound complications, including sternal instability. Medical history will be taken to establish hospital readmissions and accident & emergency department attendance for surgery-related complications. If a participant misses this appointment, they will be sent another.

### Data collection

At pre-CR and post-CR appointments and the final follow-up assessment, participants will be asked to complete quality of life (EQ-5D-5L) and resource use questions (see table 1). If a participant does not attend, the questionnaire will be posted out to them, with two reminders subsequently sent if no response (1 and 2 weeks later as necessary).

**Table 1:** Study assessment schedule

			Control arm					Intervention arm				
Procedure	Surgery	Eligibility/ discharge	Outpatient appointment	Pre-CR	CR sessions (8–16 sessions over 8 weeks)	Post-CR	Final follow-up visit	Outpatient appointment	Pre-CR	CR sessions (8–16 sessions over 8 weeks)	Post-CR	Final follow-up visit
	Week −1	Week 0	Week 6^#^	Week 8		Week 16	Week 26	Week 3	Week 4		Week 12	Week 26
Surgery	·*											
Screening for eligibility		·										
Consent		·										
Baseline data collection		·										
Randomisation		·										
Clinical examination		·*	·*				·	·*				·
Cardiopulmonary exercise test		·†					·†					·†
Incremental shuttle walk test				·		·	·		·		·	·
EQ-5D-5L		·		·		·	·		·		·	·
CR				·*		·*			·*		·*	
Vital signs—heart rate, blood pressure, oxygen saturation		·*	·*	·*				·*	·*			
Participant interviews‡			·				·	·				·
Session attendance					·					·	
Session content					·					·	
Participant diary					·				·		·
Resource use questions				·		·	·		·		·	·
Adverse event monitoring			·*	·		·	·	·*	·		·	·

*Part of normal patient pathway.

†For only 25 participants in each group from Hull site.

‡For a purposive sample of up to 10 patients in each treatment arm.

CR, cardiac rehabilitation; EQ-5D-5L, EuroQol-5 dimensions-5 levels.

### Qualitative data collection

After the outpatient review, we will conduct semistructured interviews by telephone with approximately 10 participants from each treatment arm (20 in total) to determine their views on the timing of their postoperative outpatient review and readiness to commence CR. These participants will be purposively selected from those who agreed to be approached for interview. A second interview will be held with the same participants on their completion of the study to determine their views on the timing of the outpatient review and CR, study conduct and processes. All interviews will be digitally recorded with participant permission.

Participants will be encouraged to complete bespoke CR diaries for the 8 weeks of CR detailing their experience at each session, including willingness to take part and any difficulties.

Research nurses will complete a diary detailing recruitment of patients, reasons for non-consent and any difficulties encountered with patient follow-up. CR staff will be asked to keep a diary collecting information about participant drop-outs/opt-outs, level of participation and adverse events (AEs) during the sessions. In addition, staff involved in the research will be invited a focus group at the end of final follow-up to discuss any difficulties encountered with recruitment, outpatient review, CR sessions and follow-up. Organisational and clinical barriers will also be examined.

Data will be analysed separately for patients, research staff and clinical staff using conventional content analysis.^33^ Transcribed data from the interviews and focus groups will be downloaded into qualitative data analysis computer software package, coded and analysed inductively using thematic content analysis^34^ to inform the development of the logistical processes involved in delivering a large-scale multicentre RCT.

### Statistical analyses

A full statistical analysis plan detailing intended analyses will be drafted before completion of data collection. Analysis and reporting will follow Consolidated Standards of Reporting Trials guidelines^35 36^ and a flow diagram will depict the progression of participants through the trial. Baseline data will be summarised by randomised group with no formal statistical comparisons undertaken. Continuous variables will be summarised using descriptive statistics (n, mean, SD, median, minimum and maximum) while categorical data will be reported as counts and percentages. Participant outcomes will be summarised descriptively by randomised group and time point, including extent of missing data. As this is a feasibility trial, formal hypothesis testing for effectiveness will not be carried out. The number of participants attending their review appointment and entering CR, and the time between surgery and these events, will be summarised by treatment group. Questionnaire return rates will be presented and AEs will be summarised descriptively. No interim analyses will be conducted.

### Health economics analysis

A full cost-effectiveness analysis will not be undertaken as part of this feasibility study. Instead, the study will consider the feasibility of collecting the data needed for an economic analysis of a full-scale trial and explore the rate of response and missingness of relevant questionnaires. Estimates of patient benefit, determined from the EQ-5D-5L, and NHS resource use, using patient reported questionnaires, will be summarised for the two arms of the trial. Mortality and AE rates will also be monitored to determine the importance of long-term extrapolation of related outcomes for any future analysis of a full-scale RCT. As this is a feasibility trial, formal estimation of the cost-effectiveness of the respective interventions will not be carried out.

### Blinding

By the nature of the timing of the study treatments, blinding of the participants and clinicians is not possible and a procedure for unblinding is not necessary. The clinician evaluating the CPET data will be blinded. The statistician and health economist will be blinded to study allocation.

### Safety measurements

AEs and serious AEs (SAEs) will be recorded throughout the study. Intensity and relationship to the study intervention will be described. Examples of AE related to participation in this study would be: atrial fibrillation, sternal pain and undue fatigue or exhaustion. An example of an SAE would be sternal instability.

Participants will undergo clinical assessment after discharge and be certified suitable for CR by the surgical team. Before commencing CR, they will undergo exercise testing, and CR will be tailored to their fitness levels. At completion of CR, they will have another assessment and exit consultation.

Information about AEs whether patient reported, discovered via clinical team questioning, identified through physical examination or in medical notes, will be recorded up to and including final follow-up. Ongoing review of AEs will take place during monthly trial management group (TMG) meetings, discussed with the patient advisory group (PAG) and trial steering committee (TSC) and reported to the sponsor and research ethics committee in line with their guidelines.

### Data collection, integrity and management

Data collected as part of this research includes questionnaires, clinical assessments, information from medical records and qualitative data from interviews. Data will be collected through designed questionnaires identified by a unique participant trial number only. Each site will hold data according to the current Data Protection Act (2018)^37^ and the General Data Protection Regulation (May 2018).^38^

All information collected during the study will be kept strictly confidential and stored on a secure password-protected server located at the University of York, for the purposes of assisting in follow-ups during the study. All paper documents will be stored securely. Electronic sound files and transcripts from qualitative interviews will be assigned a unique participant number, known only to the qualitative researcher and appropriate members of the research team. Any quotes published will be anonymous. All data will be archived for 10 years following the end of the study.

### Patient and public involvement

A PAG will hold meetings with research nurses, CR staff and sites’ principal investigators during the study to discuss any challenges encountered and how to overcome them. The group act as an important source of reference and the research progress will be discussed. Their role initially included assisting with the development of study documentation ensuring material was clear to the lay public; identifying barriers to participation and possible mitigation of those barriers; and providing input for topic guides for the qualitative research. Towards completion of the study, the PAG will identify appropriate pathways for dissemination and contribute to writing the lay summary.

## Ethics, oversight and dissemination

The TMG, led by the chief investigator, are responsible for overseeing the day-to-day running and management of the trial with YTU responsible for project management. Due to the low-risk nature of this trial, one independent steering and monitoring committee will fulfil the roles traditionally undertaken by the TSC and data monitoring committee. It will comprise of a chair, statistician, one other independent member and public involvement representative.

Study findings will be discussed with participants, PAG, and at a meeting of local general practitioners. Study results will be presented at annual conferences of The Society for Cardiothoracic Surgery of Great Britain and Ireland, BACPR and The European Association for Cardiothoracic Surgery. Study results will also be submitted to the funders and peer-reviewed journals.

## Supplementary Material

Reviewer comments

Author's manuscript
