# Supplementary material for: Feasibility study of early outpatient review and early cardiac rehabilitation after cardiac surgery: mixed-methods research design—a study protocol
Source: BMJ Open. 2019 Dec 29;9(12):e035787. doi: 10.1136/bmjopen-2019-035787 (PMC6937080; doi:10.1136/bmjopen-2019-035787)
Supplement: Author's manuscript [file bmjopen-2019-035787.draft_revisions.pdf]

BMJ Open

BMJ Open is committed to open peer review. As part of this commitment we make the peer review history of every article we publish publicly available.

When an article is published we post the peer reviewers' comments and the authors' responses online. We also post the versions of the paper that were used during peer review. These are the versions that the peer review comments apply to.

The versions of the paper that follow are the versions that were submitted during the peer review process. They are not the versions of record or the final published versions. They should not be cited or distributed as the published version of this manuscript.

BMJ Open is an open access journal and the full, final, typeset and author-corrected version of record of the manuscript is available on our site with no access controls, subscription charges or pay-per-view fees (http://bmjopen.bmj.com).

If you have any questions on BMJ Open's open peer review process please email info.bmjopen@bmj.com

BMJ Open

Feasibility Study of Early Outpatient Review and Early Cardiac Rehabilitation After Cardiac Surgery: Mixed Methods Research Design – a study protocol

Journal:	*BMJ Open*
Manuscript ID	bmjopen-2019-035787
Article Type:	Protocol
Date Submitted by the Author:	15-Nov-2019

Complete List of Authors:	Ngaage, Dumbor; Hull University Teaching Hospitals NHS Trust, Castle Hill Hospital<br>Mitchell, Natasha; University of York, York Trials Unit, Department of Health Sciences<br>Dean, Alexandra; University of York, York Trials unit, Department of Health Sciences<br>Hirst, Claire; University of York, York Trials Unit, Department of Health Sciences<br>Akowuah, Enoch; South Tees Hospitals NHS Foundation Trust, James Cook Hospital<br>Doherty, Patrick Joseph; University of York, Department of Health Sciences<br>Fairhurst, Caroline; University of York, York Trials unit, Department of Health Sciences<br>Flemming, Kate; University of York, Department of Health Sciences<br>Hewitt, Catherine; The University of York, York Trials Unit, Department of Health Sciences<br>Hinde, Sebastian; University of York, Centre for Health Economics<br>Mitchell, Alex; University of York, York Trials Unit, Department of Health Sciences<br>Nichols, Simon; Sheffield Hallam University - Collegiate Crescent Campus, Centre for Sport and Exercise Science<br>Watson, Judith; University of York, York Trials Unit, Department of Health Sciences

Keywords:	REHABILITATION MEDICINE, Cardiothoracic surgery < SURGERY, STATISTICS & RESEARCH METHODS, HEALTH ECONOMICS

SCHOLARONE™
Manuscripts

Feasibility Study of Early Outpatient Review and Early Cardiac Rehabilitation After Cardiac Surgery: Mixed Methods Research Design – a study protocol

Authors:

Dumbor L Ngaage[1], Natasha Mitchell[2], Alexandra Dean[2], Claire Hirst[2], Enoch Akowuah[3], Patrick Doherty[2], Caroline Fairhurst[2], Kate Flemming[2], Catherine Hewitt[2], Sebastian Hinde[4], Alex Mitchell[2], Simon Nichols[5], Judith Watson[2]

Corresponding author: Mr Dumbor L Ngaage - Castle Hill Hospital, Hull University Teaching Hospitals NHS Trust, Castle Road Cottingham, Hull, UK; dumbor.ngaage@hey.nhs.uk; 01482623263

Author affiliations:

1. Castle Hill Hospital, Hull University Teaching Hospitals NHS Trust, Castle Road Cottingham, Hull, UK
   dumbor.ngaage@hey.nhs.uk - Orcid iD 0000-0003-2378-7160
2. Department of Health Sciences, University of York, ARRC Building, York, UK
   natasha.mitchell@york.ac.uk - Orcid iD 0000-0002-6904-2976
   alexandra.dean@york.ac.uk- Orcid iD 0000-0003-4711-5609
   claire.hirst@york.ac.uk - Orcid iD 0000-0001-7832-5030
   patrick.doherty@york.ac.uk Orcid iD 0000-0002-1887-0237
   caroline.fairhurst@york.ac.uk - Orcid iD 0000-0003-0547-462X
   kate.flemming@york.ac.uk - Orcid iD 0000-0002-0795-8516
   catherine.hewitt@york.ac.uk - Orcid iD 0000-0002-0415-3536
   alex.mitchell@york.ac.uk - Orcid iD 0000-0001-9311-2092
   jude.watson@york.ac.uk- Orcid iD 0000-0003-0694-3854
3. James Cook Hospital, South Tees Hospitals NHS Foundation Trust, Marton Road Middlesbrough, UK
   enoch.akowuah@nhs.net - Orcid iD 0000-0002-2429-3579
4. Centre for Health Economics, Alcuin A Block, University of York, York UK
   sebastian.hinde@york.ac.uk- Orcid iD 0000-0002-7117-4142
5. Centre for Sport and Exercise Science, Sheffield Hallam University, Collegiate Campus, Sheffield, UK
   S.J.Nichols@shu.ac.uk - Orcid iD 0000-0003-0377-6982

Main text word count: 4346

For peer review only - http://bmjopen.bmj.com/site/about/guidelines.xhtml

Abstract

Background: Following cardiac surgery, patients currently attend an outpatient review six weeks after hospital discharge, where recovery is assessed and suitability to commence cardiac rehabilitation (CR) is determined. CR is then started from eight weeks. Following a median sternotomy, cardiac surgery patients are required to refrain from upper body exercises, lifting of heavy objects and other strenuous activities for 12 weeks. A delay in starting CR can prolong the recovery process, increase dependence on family/carers and can cause frustration. However, current guidelines for activity and exercise after median sternotomy have been described as restrictive, anecdotal and increasingly at odds with modern clinical guidance for CR. This study aims to examine the feasibility of bringing forward outpatient review and starting CR earlier.

Methods and analyses: This is a multi-centre, randomised controlled, open feasibility trial comparing postoperative outpatient review 6 weeks after hospital discharge, followed by CR commencement from 8 weeks (control arm) versus, postoperative outpatient review 3 weeks after hospital discharge, followed by commencement of CR from 4 weeks (intervention arm). The study aims to recruit 100 eligible patients, aged 18 to 80 years who have undergone elective or urgent cardiac surgery involving a full median sternotomy, over a 7 month period across two centres. Feasibility will be measured by consent, recruitment, retention rates, and attendance at appointments and CR sessions. Qualitative interviews with trial participants and staff will explore issues around study processes and acceptability of the intervention. Findings from the qualitative work will be integrated with the feasibility trial outcomes to inform the design of a future full-scale RCT.

Ethics and dissemination: Ethics approval was granted by East Midlands - Derby Research Ethics Committee on 10th January 2019. The findings will be presented at relevant conferences disseminated via peer-reviewed research publications, and to relevant stakeholders.

Trial registration: ISRCTN80441309

Keywords: cardiac surgery; cardiac rehabilitation; outpatient review; feasibility; median sternotomy

Protocol version: Version 2.0, 26th September 2019

For peer review only - http://bmjopen.bmj.com/site/about/guidelines.xhtml

ARTICLE SUMMARY

Strengths and limitations of this study

- Currently, there is a lack of published data about the appropriate timing of post-operative review and cardiac rehabilitation following cardiac surgery.
- This feasibility study is a small multi-centre RCT.
- The study will collect both qualitative and quantitative data.
- If it is determined that a larger scale randomised controlled trial is feasible, this study will generate valuable data to enable its design.
- This feasibility study is not large enough to determine effectiveness or cost-effectiveness.

For peer review only

INTRODUCTION

Following cardiac surgery, patients currently attend their first outpatient review six weeks after hospital discharge, where recovery is assessed and ability to commence cardiac rehabilitation (CR) is determined. CR is then started from eight weeks. In a 2017 survey, 35 of the 42 UK cardiac centres who responded, confirmed this as current practice [1]. This interval before review and CR extends the period of inactivity of patients, and medical attention for surgery-related complications is often sought during this period [2,3,4]. Our prospective observational study (FORCAST6) found that 39% of patients reported surgery-related complications in the six weeks following discharge, with the majority occurring in the first 4 weeks; 15% required hospital readmission. Although the majority of patients were satisfied with the six weeks interval, 44% felt it was too long and would have liked an earlier review [1].

The standard access for heart operations is through a median sternotomy [5,6,7,8]. In the UK, 35,158 heart operations were performed in 2015 [9]. Following a median sternotomy, patients are required to refrain from upper body exercises, lifting of heavy objects and other strenuous activities for 12 weeks [10,11,12,13]. These "sternal precautions" (SP) are intended to aid healing of the sternum. CR, which has significant short- and long-term benefits after cardiac surgery [14,15], is therefore delayed. The delay can mitigate the benefits of CR [16], contributing to physical deconditioning, and hinder the ability of CR to facilitate timely recovery of fitness and physical activity status [17]. However, current guidelines for activity and exercise after median sternotomy have been described as restrictive, conflicting, sometimes arbitrary, frequently anecdotal and frequently at odds with modern clinical guidance [11].

Following surgery, there are variations in types of exercises permitted, limits for weight of objects that can be lifted, guidelines for activities, and timeline for resumption of driving. Parker et al [18] demonstrated that the force elicited on the breastbone by coughing far exceeded lifting above the recommended limit. Adams and colleagues [11] investigated forces associated with 32 activities of daily living and reported that the majority not restricted by SP, such as opening and closing doors, generated forces greater than the allowed weight limit. While SP may help to support bone healing, the optimal nature and duration are unclear especially since sternal bone healing occurs by five weeks [12]. According to the 2013 UK national audit of CR, late commencement contributes to a substantial number of heart

For peer review only - http://bmjopen.bmj.com/site/about/guidelines.xhtml

surgery patients declining to participate [19]. Adverse outcomes following heart operations and other forms of surgery have been shown to be reduced by early patient review after hospital discharge [20,21]. The FORCAST6 study showed a high incidence of postoperative complications with the current 6-week patient review after heart surgery. This was highest in the first week after hospital discharge and declined to lowest levels by 4 weeks [1]. It therefore seems logical to conduct patient reviews after hospital discharge sooner than current practice to enable early CR from the period of postoperative stability and sternal bone healing at 5 weeks after surgery (4 weeks after hospital discharge).

The British Association for Cardiovascular Prevention and Rehabilitation (BACPR) Standards and Core Components [22] and the National Certification Programme for CR [23] recommend early commencement of CR. This is monitored by the National Audit of CR 2018 [19**Error! Bookmark not defined.**]. A delay in starting CR can prolong the recovery process, increase dependence on family/carers and can cause frustration. This may contribute to anxiety and depression that is reported in patients recovering after cardiac surgery [24].

This study aims to examine the feasibility of bringing forward outpatient review and CR in order to facilitate recovery, physical fitness and quality of life.

METHODS AND ANALYSIS

Study design

A multi-centre, randomised controlled, open feasibility trial using mixed methods to establish the feasibility of conducting a study where:

- Participants have a postoperative outpatient review 6 weeks after hospital discharge, followed by commencement of CR from 8 weeks (*control arm*); or

- Participants have a postoperative outpatient review 3 weeks after hospital discharge, followed by commencement of CR from 4 weeks (*intervention arm*).

The study is detailed in Figure 1: Study Flowchart.

For peer review only - http://bmjopen.bmj.com/site/about/guidelines.xhtml

Study aim

To establish the feasibility of delivering and evaluating a definitive RCT of the current 8th week versus 4th week CR commencement pathways, reducing the period over which patients are required to refrain from usual physical activities following cardiac surgery.

Study objectives

The primary objective is to determine the feasibility of delivering outpatient review three weeks after discharge post-cardiac surgery, followed by CR from four weeks.

Secondary objectives

- Assess surgeons' and surgical practitioners' willingness to conduct outpatient review three weeks after discharge and refer patients to CR.
- Examine barriers to patient enrolment.
- Identify recruitment rates and drop-out to follow-up.
- Identify the most appropriate outcome measures.
- Test follow-up procedures and data collection tools and management.
- Assess the feasibility of conducting an economic evaluation of any future definitive RCT.
- Gather outcome data for power and sample size calculations for the RCT.
- Inform any necessary redesign of a new recovery pathway in light of information gained.

Study population and setting

Patients attending for cardiac surgery at Hull University Teaching Hospitals NHS Trust, UK and South Tees Hospitals NHS Foundation Trust, UK will be approached regarding study participation.

Inclusion criteria

- Undergone elective or urgent cardiac surgery, including the following procedures (isolated or combined):
    - Coronary artery bypass graft (CABG)

For peer review only - http://bmjopen.bmj.com/site/about/guidelines.xhtml

For peer review only

- Aortic valve replacement
- Mitral valve repair or replacement
- Atrial fibrillation ablation
- Left atrial appendage occlusion

– Has had a full median sternotomy

– Willing and capable of giving informed consent

– Aged 18 to 80 years at the time of giving consent

– Able to self-complete the English language outcome measure tools (or can complete with assistance)

– Able to follow detailed verbal instructions required for clinical assessments.

Exclusion criteria

– Body mass index (BMI) >45kg/m^2

– Heart failure with left ventricular ejection fraction of <30% before surgery

– Early postoperative sternal wound complications (e.g. deep sternal wound infection, wound dehiscence and/or sternal instability)

– Postoperative complications resulting in prolonged hospital stay >14 days after surgery.

For peer review only

Amendment to eligibility criteria

During the initial recruitment period, fewer patients than anticipated were meeting the eligibility criteria, and many were 'out of area' impacting on their ability to attend study CR locations. With the funder's approval, a substantial amendment was submitted to the ethics committee. The original criteria had only included patients who had undergone CABG, had an upper age limit of 75 years and a maximum BMI limit of 40kg/m^2. This amendment was approved on 30th October 2019.

Sample size

For peer review only - http://bmjopen.bmj.com/site/about/guidelines.xhtml

This study is a feasibility RCT and therefore does not have a primary outcome measure to inform a power calculation. Sample sizes of between 24 and 70 have been recommended for feasibility trials to allow for the reliable estimation of a standard deviation for use in future sample size calculations [25,26]. The plan is to recruit 100 eligible patients, allowing for a 30% attrition rate in order to still have 70 patients in the final analysis. There will be a 7 month recruitment period across two centres.

Participant recruitment

Patients scheduled for elective cardiac surgery will be given the study leaflet prior to surgery. Patients undergoing urgent cardiac surgery will receive it following surgery. All patients will undergo surgery as normal, receiving standard in-hospital postoperative care.

Eligibility and consent

After discharge from intensive care to the ward following surgery, the medical staff will review patients' routine clinical examination results, including sternal stability and postoperative tests. Those identified as potentially eligible, interested and willing, will have their eligibility confirmed by the Research Nurse using an eligibility checklist and written informed consent will be obtained according to GCP guidelines. Consent will also be sought to contact participants who are interested in being interviewed as part of an embedded qualitative study.

Patients screened as eligible, but who develop complications between being considered ready for discharge and going home, will not proceed to consent until deemed well enough (if remain in hospital for >14 days after surgery, as per exclusion criteria they will not proceed at all).

Randomisation

Following surgery, consent, and completion of baseline data collection and assessments, participants will be randomised to either the control or intervention arm.

For peer review only - http://bmjopen.bmj.com/site/about/guidelines.xhtml

Randomisation will be performed by a remote, centralised randomisation service provided by York Trials Unit (YTU), and the allocation sequence generated by a statistician not involved in the study. Participants will be individually randomised and stratified according to study site on a 1:1 basis using variable block sizes. Authorised site research staff will telephone the randomisation service to obtain the participant's allocation.

Outcomes

Participant self-reported data will be collected at baseline, prior to CR commencement, following CR completion and at final follow-up assessment (up to 26 weeks post-randomisation):

i. Demographics (baseline only)
ii. EQ-5D-5L: a validated generic patient-reported outcome measure. [27]
iii. NHS resource use.

The following clinical data will be collected:

i) height, weight, BMI, pre-operative presentation (baseline only);
ii) heart rate, blood pressure, oxygen saturation at baseline, outpatient review and pre-CR as per usual practice;
iii) physical fitness assessed by dynamic testing with incremental shuttle walk test (ISWT) for both study arms at commencement and end of CR, and at final follow-up;
iv) cardiopulmonary fitness assessed by cardiopulmonary exercise testing at baseline and at final follow-up, for up to 25 patients in each study arm [Hull study site only];
v) 30- and 90-day mortality, surgical site complications and hospital readmission rates.

In line with the objectives of a feasibility study, we will also gather information on:

vi) recruitment rates and drop-out to follow-up;
vii) compliance to treatment arm allocation;
viii) acceptability of patient recruitment, early outpatient review and CR to patients, clinicians and NHS organisations.

Cardiopulmonary exercise testing (CPET)

For peer review only

Maximal CPET provides a holistic assessment of the cardiovascular, ventilatory and metabolic responses to exercise and is a powerful diagnostic and prognostic tool [28]. Maximal CPET is the gold-standard method of assessing changes in aerobic fitness which is potentially important if we are to assess the beneficial/not harmful effects of early CR. Maximal CPET will be conducted on a cycle ergometer with patients pedalling at 50 rpm throughout the test. The test workload will start at 0w and increase in 10w/min increments until the patient reaches volitional exhaustion (ideally 8-12 minutes [28]). Maximal CPET will only be carried out (after randomisation) in up to 25 participants in each arm in the Hull site only due to costs and logistics, but also to ensure that test procedures and data interpretation are consistent. CPET will be completed once a participant is deemed clinically stable and conducted within 14 days after surgery. It will be repeated at the final follow-up.

Postoperative outpatient review

As standard practice, all participants will have a postoperative outpatient clinical review in order to be certified suitable to commence CR. This clinical decision is usually based on the absence of limiting complications. This review will take place at six weeks post-hospital discharge in the control arm, and three weeks post-discharge in the intervention arm. Participants considered unfit for CR will be given a second review appointment for approximately one week later and if necessary, a third review appointment two weeks later. Reason(s) they were deemed unfit will be documented. If they are deemed unfit for CR at the third review, they will not commence CR as part of the FARSTER study. They will, however, continue to be monitored as per usual practice and be sent all study questionnaires for completion.

Pre-cardiac rehabilitation assessment

Referred participants will be offered a comprehensive programme and an assessment prior to starting their programme. Participants will receive a holistic assessment from a specialist physiotherapist or exercise professional including exercise testing using the ISWT to help personalise their exercise prescription. The ISWT is a popular aerobic fitness assessment tool in UK CR programmes and recommended by the CR physiotherapist association [29, 30]. Although reference values for ISWT are available in conventional CR patient populations [31],

For peer review only - http://bmjopen.bmj.com/site/about/guidelines.xhtml

they have not yet been validated for use in the early CR cohorts. If a participant misses this appointment they will be sent another.

Cardiac rehabilitation programme

This will consist of supervised low-to-moderate intensity exercise performed once (South Tees) or twice (Hull) a week for eight weeks (as is standard UK practice). Exercise will be prescribed according to standards published by BACPR [22]. Attendance at these sessions and session content will be recorded for each participant.

Post-cardiac rehabilitation appointment

Following completion of the CR programme (16 weeks post-discharge for the control arm and 12 weeks post-discharge for the intervention arm), all participants will have a repeat ISWT. If a participant misses this appointment they will be sent another. A discharge letter will be sent to participants' General Practitioners summarising their treatment.

Final follow up assessment

At final follow-up (up to 26 weeks after randomisation), participants will have a repeat CPET (if appropriate), undergo a final ISWT, have an end-of-study consultation and be examined for sternal wound complications, including sternal instability. Medical history will be taken to establish hospital readmissions and Accident & Emergency department attendance for surgery-related complications. If a participant misses this appointment they will be sent another.

DATA COLLECTION

At pre-CR and post-CR appointments and the final follow up assessment, participants will be asked to complete quality of life (EQ-5D-5L) and resource use questions (see Table 1: Study Assessment Schedule). If a participant does not attend, the questionnaire will be posted out to them, with two reminders subsequently sent if no response (one and two weeks later as necessary).

For peer review only - http://bmjopen.bmj.com/site/about/guidelines.xhtml

Table 1: Study assessment schedule

			Control arm					Intervention arm				
**Procedure**	**Surgery**	**Eligibility/ Discharge**	**Outpatient appointment**	**Pre-CR**	**CR sessions** (8-16 sessions over 8 weeks)	**Post-CR**	**Final follow up visit**	**Outpatient appointment**	**Pre-CR**	**CR sessions** (8-16 sessions over 8 weeks)	**Post-CR**	**Final follow up visit**
	Week -1	*Week 0*	*Week 6#*	*Week 8*		*Week 16*	*Week 26*	*Week 3*	*Week 4*		*Week 12*	*Week 26*
Surgery	●*											
Screening for eligibility		●										
Consent		●										
Baseline data collection		●										
Randomisation		●										
Clinical examination		●*	●*				●	●*				●
Cardiopulmonary exercise test		●^					●^					●^
Incremental shuttle walk test				●		●	●		●		●	●
EQ-5D-5L		●		●		●	●		●		●	●
Cardiac rehabilitation				●*		●*			●*		●*	
Vital signs – heart rate, blood pressure, oxygen saturation		●*	●*	●*				●*	●*			
Participant interviews+			●				●	●				●
Session attendance					●					●		
Session content					●					●		
Participant diary					●				●		●	
Resource use questions				●		●	●		●		●	●
Adverse event monitoring			●*	●		●	●	●*	●		●	●

* part of normal patient pathway; ^ for only 25 participants in each group from Hull site; + for a purposive sample of up to 10 patients in each treatment arm

For peer review only

For peer review only - http://bmjopen.bmj.com/site/about/guidelines.xhtml

QUALITATIVE DATA COLLECTION

After the outpatient review, we will conduct semi-structured interviews by telephone with approximately ten participants from each treatment arm (20 in total) to determine their views on the timing of their postoperative outpatient review and readiness to commence CR. These participants will be purposively selected from those who agreed to be approached for interview. A second interview will be held with the same participants on their completion of the study to determine their views on the timing of the outpatient review and CR, study conduct and processes. All interviews will be digitally recorded with participant permission.

Participants will be encouraged to complete bespoke CR diaries for the eight weeks of CR detailing their experience at each session, including willingness to take part and any difficulties.

Research Nurses will complete a diary detailing recruitment of patients, reasons for non-consent, and any difficulties encountered with patient follow-up. CR staff will be asked to keep a diary collecting information about participant drop-outs/opt-outs, level of participation and adverse events during the sessions. In addition, staff involved in the research will be invited a focus group at the end of final follow-up to discuss any difficulties encountered with recruitment, outpatient review, CR sessions and follow-up. Organisational and clinical barriers will also be examined.

Data will be analysed separately for patients, research staff and clinical staff using conventional content analysis [32]. Transcribed data from the interviews and focus groups will be downloaded into qualitative data analysis computer software package, coded and analysed inductively using thematic content analysis [33] to inform the development of the logistical processes involved in delivering a large-scale multi-centre RCT.

STATISTICAL ANALYSES

For peer review only

A full statistical analysis plan detailing intended analyses will be drafted before completion of data collection. Analysis and reporting will follow CONSORT guidelines [34,35] and a flow diagram will depict the progression of participants through the trial. Baseline data will be summarised by randomised group with no formal statistical comparisons undertaken. Continuous variables will be summarised using descriptive statistics (n, mean, standard deviation, median, minimum and maximum) while categorical data will be reported as counts and percentages. Participant outcomes will be summarised descriptively by randomised group and time point, including extent of missing data. As this is a feasibility trial, formal hypothesis testing for effectiveness will not be carried out. The number of participants attending their review appointment and entering CR, and the time between surgery and these events, will be summarised by treatment group. Questionnaire return rates will be presented and adverse events will be summarised descriptively. No interim analyses will be conducted.

HEALTH ECONOMICS ANALYSIS

A full cost-effectiveness analysis will not be undertaken as part of this feasibility study. Instead, the study will consider the feasibility of collecting the data needed for an economic analysis of a full scale trial and explore the rate of response and missingness of relevant questionnaires. Estimates of patient benefit, determined from the EQ-5D-5L, and NHS resource use, using patient reported questionnaires, will be summarised for the two arms of the trial. Mortality and adverse event rates will also be monitored to determine the importance of long term extrapolation of related outcomes for any future analysis of a full scale RCT. As this is a feasibility trial, formal estimation of the cost-effectiveness of the respective interventions will not be carried out.

BLINDING

By the nature of the timing of the study treatments, blinding of the participants and clinicians is not possible and a procedure for unblinding is not necessary. The clinician evaluating the CPET data will be blinded. The statistician and health economist will be blinded to study allocation.

For peer review only - http://bmjopen.bmj.com/site/about/guidelines.xhtml

SAFETY MEASUREMENTS

Adverse events (AEs) and serious adverse events (SAE), will be recorded throughout the study. Intensity and relationship to the study intervention will be described. Examples of AE related to participation in this study would be: atrial fibrillation; sternal pain, and; undue fatigue or exhaustion. An example of a SAE would be sternal instability.

Participants will undergo clinical assessment after discharge and be certified suitable for CR by the surgical team. Before commencing CR they will undergo exercise testing, and CR will be tailored to their fitness levels. At completion of CR, they will have another assessment and exit consultation.

Information about AEs whether patient reported, discovered via clinical team questioning, identified through physical examination or in medical notes, will be recorded up to and including final follow-up. Ongoing review of AEs will take place during monthly Trial Management Group (TMG) meetings, discussed with the Patient Advisory Group (PAG) and Trial Steering Committee (TSC) and reported to the sponsor and Research Ethics Committee in line with their guidelines.

DATA COLLECTION, INTEGRITY AND MANAGEMENT

Data collected as part of this research includes questionnaires, clinical assessments, information from medical records and qualitative data from interviews. Data will be collected through designed questionnaires identified by a unique participant trial number only. Each site will hold data according to the current Data Protection Act (2018) [36] and the General Data Protection Regulation (May 2018) [37].

All information collected during the study will be kept strictly confidential and stored on a secure password-protected server located at the University of York, for the purposes of assisting in follow-ups during the study. All paper documents will be stored securely. Electronic sound files and transcripts from qualitative interviews will be assigned a unique participant number, known only to the qualitative researcher and appropriate members of

For peer review only

the research team. Any quotes published will be anonymous. All data will be archived for ten years following the end of the study.

PATIENT AND PUBLIC INVOLVEMENT

A PAG will hold meetings with research nurses, CR staff and sites' Principal investigators during the study to discuss any challenges encountered and how to overcome them. The group act as an important source of reference and the research progress will be discussed. Their role initially included assisting with the development of study documentation ensuring material was clear to the lay public; identifying barriers to participation and possible mitigation of those barriers; and providing input for topic guides for the qualitative research. Towards completion of the study, the PAG will identify appropriate pathways for dissemination and contribute to writing the lay summary.

ETHICS, OVERSIGHT AND DISSEMINATION

The TMG, led by the Chief Investigator, are responsible for overseeing the day-to-day running and management of the trial with YTU responsible for project management. Due to the low risk nature of this trial, one independent steering and monitoring committee will fulfil the roles traditionally undertaken by the TSC and Data Monitoring Committee. It will comprise of a Chair, statistician, one other independent member, and Public Involvement representative.

Study findings will be discussed with participants, PAG, and at a meeting of local General Practitioners. Study results will be presented at annual conferences of The Society for Cardiothoracic Surgery of Great Britain and Ireland, BACPR, and The European Association for Cardiothoracic Surgery. Study results will also be submitted to the funders and peer-reviewed journals.

CONCLUSION

Current time frames for postoperative review and CR are not evidence-based and may prolong recovery following cardiac surgery, placing unnecessary limitations on patient activity. Feedback and findings from this feasibility study would be used to design a

For peer review only - http://bmjopen.bmj.com/site/about/guidelines.xhtml

definitive multi-centre RCT which would determine the clinical and cost benefits of the proposed recovery pathway.

Figure legend

Figure 1: FARSTER study flowchart

Acknowledgements

The authors would like to thank Carmen Neave and Karen Dobbs, the Research Nurses at the sites; all the clinical and CR staff at the participating sites; and the members of the PAG.

Author Contributions

DN, PD and SN led on the conception, design and writing of the study. DN led the grant application and as Chief Investigator, takes overall responsibility for the trial. JW led on the writing of the study protocol with substantial contributions to the design, writing, critical review of intellectual content and final manuscript approval from DN, NM, AD, CHi, EA, PD, CF, KF, CHe, SB, AM and SN. CF, CHe and AM provided statistical expertise in the study design and development stages of the project and the protocol. CHi, AD, NM and JW made substantial contributions to the trial design and management. KF led on the qualitative study design. SH was specifically responsible for the health economic aspects of the study design and DN, EA, PD and SN were responsible for providing expert clinical support. All authors agree to be accountable for their work.

Funding statement

Hull University Teaching Hospitals NHS Trust are the sponsors on behalf of the funder, who is the NIHR as stated below. This paper presents independent research funded by the National Institute for Health Research (NIHR) under its Research for Patient Benefit (RfPB) Programme (Grant Reference Number PB-PG-0317-20047).

Disclaimer

The views expressed are those of the author(s) and not necessarily those of the NHS, the NIHR, or the Department of Health and Social Care.

Competing interests

None declared.

Patient consent for publication

Not required.

For peer review only - http://bmjopen.bmj.com/site/about/guidelines.xhtml

Ethics approval

Ethics approval was granted by East Midlands - Derby Research Ethics Committee on 10th January 2019 (REC Reference: 18/EM/0391).

Data availability statement

There are no data in this work, as this is a trial protocol.

For peer review only

REFERENCES

1. Ngaage DL, Gooseman M, Bulliment K, et al. Is six weeks too long for first planned postoperative review after cardiac surgery? FORCAST6. *Br J Cardiol* 2019;26:34 doi:10.5837/bjc.2019.008
2. Shroyer AL, Coombs LP, Peterson ED, et al. The Society of Thoracic Surgeons: 30-day operative mortality and morbidity risk models. *Ann Thor Surg* 2003; 75(6): 1856-64; discussion 1864-5.
3. Fox JP, Suter LG, Wang K, et al. Hospital-based, acute care use among patients within 30 days of discharge after coronary artery bypass surgery. *Ann Thorac Surg* 2013; 96(1):96-104
4. Hannan EL, Zhong Y, Lahey SJ, et al. 30-day readmissions after coronary artery bypass graft surgery in New York State. *JACC Cardiovasc Interv*. 2011; May;4(5):569-76.
5. Braimbridge MV. Median Sternotomy. *Lancet.* 1965; 1(7385):585.
6. Fischer H. [Evaluation of medial sternotomy as an approach to the heart]. *Zentralbl Chir* 1956; 81(40):2099-101.
7. Gerbode F, Braimbridge MV, Melrose DG. Median sternotomy for open cardiac surgery during total heart-lung by-pass. *AMA Arch Surg* 1958;76(5):821-3; discussion 823-4.
8. Lillehei CW, Cardozo RH. Use of median sternotomy with femoral artery cannulation in open cardiac surgery. *Surg Gynecol Obstet* 1959;108(6):706-14.
9. The Society for Cardiothoracic Surgery in Great Britain & Ireland. National Adult Cardiac Database Report. Available at http://bluebook.scts.org/. Accessed June 05, 2017.
10. British Heart Foundation. Cardiac rehabilitation. Available at www.bhf.org.uk/publications. Accessed November 26, 2017.
11. Adams J, Cline MJ, Hubbard M, et al. A new paradigm for post-cardiac event resistance exercise guidelines. *Am J Cardiol* 2006; 97(2):281-6.
12. Brocki BC, Thorup CB, Andreasen JJ. Precautions related to midline sternotomy in cardiac surgery: a review of mechanical stress factors leading to sternal complications. *Eur J Cardiovasc Nurs* 2010; 9(2):77-84.
13. Cahalin LP, Lapier TK, Shaw DK. Sternal Precautions: Is It Time for Change? Precautions versus Restrictions - A Review of Literature and Recommendations for Revision. *Cardiopulm Phys Ther* J 2011; 22(1):5-15.
14. Anderson L, Thompson DR, Oldridge N, et al. Exercise-based cardiac rehabilitation for coronary heart disease (Review). *Cochrane Database of Systematic Reviews* 2016, Issue 1. Art. No.: CD001800. DOI: 10.1002/14651858.CD001800.pub3.
15. Pack QR, Goel K, Lahr BD, et al. Participation in cardiac rehabilitation and survival after coronary artery bypass graft surgery a community-based study. *Circulation*. 2013; 128:590-7.
16. Marzolini S, Blanchard C, Alter DA, et al. Delays in Referral and Enrolment Are Associated With Mitigated Benefits of Cardiac Rehabilitation After Coronary Artery Bypass Surgery. *Circ Cardiovasc Qual Outcomes.* 2015; Nov;8(6):608-20

For peer review only - http://bmjopen.bmj.com/site/about/guidelines.xhtml

17. LaPier TK. Functional status of patients during subacute recovery from coronary artery bypass surgery. *Heart Lung* 2007; 36(2):114-24
18. Parker R, Adams JL, Ogola G, et al. Current activity guidelines for CABG patients are too restrictive: comparison of the forces exerted on the median sternotomy during a cough vs. lifting activities combined with valsalva maneuver. *Thorac Cardiovasc Surg* 2008;56(4):190-4.
19. NACR 2018. British Heart Foundation National Audit of Cardiac Rehabilitation. Quality and Outcomes Report. https://www.bhf.org.uk/informationsupport/publications/statistics/national-audit-of-cardiac-rehabilitation-quality-and-outcomes-report-2018
20. Hall MH, Esposito RA, Pekmezaris R, et al. Cardiac surgery nurse practitioner home visits prevent coronary artery bypass graft readmissions. *Ann Thorac Surg*. 2014; May;97(5):1488-93; discussion 1493-5
21. Saunders RS, Fernandes-Taylor S, Rathouz PJ, et al. Outpatient follow-up versus 30-day readmission among general and vascular surgery patients: a case for redesigning transitional care. *Surgery*. 2014; Oct;156(4):949-56.
22. BACPR. Standards and Core Components for Cardiovascular Disease Prevention and Rehabilitation. 2017. Available from: http://www.bacpr.com
23. http://www.cardiacrehabilitation.org.uk/NCP-CR.htm
24. Tully PJ, Baker RA. Depression, anxiety, and cardiac morbidity outcomes after coronary artery bypass surgery: a contemporary and practical review**.** *J Geriatric* Cardiol 2012; 9: 197−208.
25. Julious SA, Sample size of 12 per group rule of thumb for a pilot study. *Pharmaceutical Statistics*, 2005; **4**(4): p. 287-291
26. Teare MD, et al., Sample size requirements to estimate key design parameters from external pilot randomised controlled trials: a simulation study. *Trials,* 2014; **15**(1): p. 264.
27. Hurst NP, Kind P, Ruta D, et al. Measuring health-related quality of life in rheumatoid arthritis: Validity, responsiveness and reliability of EuroQol (EQ-5D). *Br J Rheumatol.* 1997; 36 (5):551-9.
28. Taylor C, Nichols S, Ingle L (2015) A clinician's guide to cardiopulmonary exercise testing 1: an introduction. *Br J Hosp Med* 76(4): 192–5 (doi: 10.12968/hmed.2015.76.4.192)
29. Grove T. Incremental shuttle walk test in cardiac rehabilitation. *B J Card Nurs,* 2013; 8:1, 31-37
30. ACPICR. Standards for physical activity and exercise in the cardiovascular population. 3rd ed. Heather P, Helen B, Samantha B, John B, Laura Burgess, Keri G, et al., editors: Association of Chartered Physiotherapists in Cardiac Rehabilitation 2015.
31. Doherty P, Alotaibi JFM. Evaluation of determinants of walking fitness in patients attending cardiac rehabilitation. *BMJ Open Sport Exerc Med* 2017;2:e000203. doi:10.1136/bmjsem-201600020
32. Hsieh, H-F, Shannon SE. Three Approaches to Qualitative Content Analysis. *Qualitative Health Research*. 2005; **15**(9): 1277-1288
33. Green J, Thorogood N. Qualitative Methods for Health Research 4th Edition. London, Sage. 2018.

For peer review only - http://bmjopen.bmj.com/site/about/guidelines.xhtml

34. Schulz KF, Altman DG, MoherD, CONSORT 2010 statement: updated guidelines for reporting parallel group randomized trials. *Ann Intern Med,* 2010; **152**(11): p. 726-32.
35. Eldridge SM, Chan CL, Campbell MJ et al., CONSORT 2010 statement: extension to randomised pilot and feasibility trials. BMJ, 2016; **355**.
36. Data Protection Act, UK Government http://www.legislation.gov.uk/ukpga/2018/12/contents/enacted
37. Information Commissioner's Office https://ico.org.uk/for-organisations/guide-to-the-general-data-protection-regulation-gdpr/

For peer review only

For peer review only - http://bmjopen.bmj.com/site/about/guidelines.xhtml

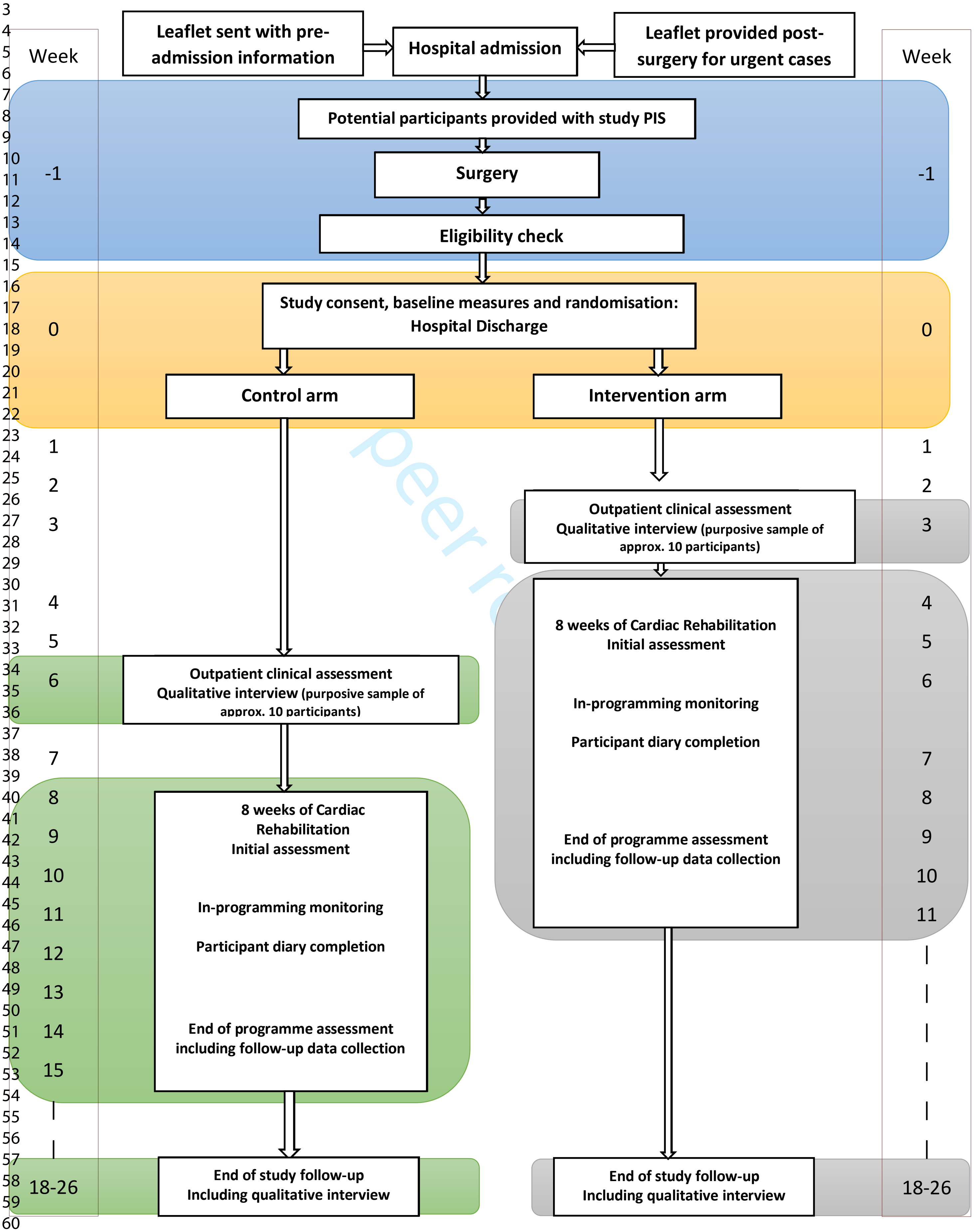
Week
Leaflet sent with pre-admission information
Hospital admission
Leaflet provided post-surgery for urgent cases
Week
Potential participants provided with study PIS
-1
Surgery
-1
Eligibility check
Study consent, baseline measures and randomisation: Hospital Discharge
0
0
Control arm
Intervention arm
1
1
2
2
3
Outpatient clinical assessment
Qualitative interview (purposive sample of approx. 10 participants)
3
4
4
5
5
8 weeks of Cardiac Rehabilitation
Initial assessment
6
Outpatient clinical assessment
Qualitative interview (purposive sample of approx. 10 participants)
6
In-programming monitoring
Participant diary completion
7
7
8
8
8 weeks of Cardiac Rehabilitation
Initial assessment
9
9
End of programme assessment including follow-up data collection
10
10
11
In-programming monitoring
11
12
Participant diary completion
13
14
End of programme assessment including follow-up data collection
15
18-26
End of study follow-up
Including qualitative interview
End of study follow-up
Including qualitative interview
18-26

Reporting checklist for protocol of a clinical trial.

Based on the SPIRIT guidelines.

Instructions to authors

Complete this checklist by entering the page numbers from your manuscript where readers will find each of the items listed below.

Your article may not currently address all the items on the checklist. Please modify your text to include the missing information. If you are certain that an item does not apply, please write "n/a" and provide a short explanation.

Upload your completed checklist as an extra file when you submit to a journal.

In your methods section, say that you used the SPIRITreporting guidelines, and cite them as:

Chan A-W, Tetzlaff JM, Altman DG, Laupacis A, Gøtzsche PC, Krleža-Jerić K, Hróbjartsson A, Mann H, Dickersin K, Berlin J, Doré C, Parulekar W, Summerskill W, Groves T, Schulz K, Sox H, Rockhold FW, Rennie D, Moher D. SPIRIT 2013 Statement: Defining standard protocol items for clinical trials. Ann Intern Med. 2013;158(3):200-207

		Reporting Item	Page Number
**Administrative information**			
Title	#1	Descriptive title identifying the study design, population, interventions, and, if applicable, trial acronym	1

For peer review only

Trial registration	#2a	Trial identifier and registry name. If not yet registered, name of intended registry	2
Trial registration: data set	#2b	All items from the World Health Organization Trial Registration Data Set	-
Protocol version	#3	Date and version identifier	2
Funding	#4	Sources and types of financial, material, and other support	17
Roles and responsibilities: contributorship	#5a	Names, affiliations, and roles of protocol contributors	1, 17
Roles and responsibilities: sponsor contact information	#5b	Name and contact information for the trial sponsor	17
Roles and responsibilities: sponsor and funder	#5c	Role of study sponsor and funders, if any, in study design; collection, management, analysis, and interpretation of data; writing of the report; and the decision to submit the report for publication, including whether they will have ultimate authority over any of these activities	17
Roles and responsibilities: committees	#5d	Composition, roles, and responsibilities of the coordinating centre, steering committee, endpoint adjudication committee, data management team, and other individuals or groups overseeing the trial, if applicable (see Item 21a for data monitoring committee)	16

For peer review only

**Introduction**			
Background and rationale	#6a	Description of research question and justification for undertaking the trial, including summary of relevant studies (published and unpublished) examining benefits and harms for each intervention	4-5
Background and rationale: choice of comparators	#6b	Explanation for choice of comparators	4-5
Objectives	#7	Specific objectives or hypotheses	6
Trial design	#8	Description of trial design including type of trial (eg, parallel group, crossover, factorial, single group), allocation ratio, and framework (eg, superiority, equivalence, non-inferiority, exploratory)	5
**Methods: Participants, interventions, and outcomes**			
Study setting	#9	Description of study settings (eg, community clinic, academic hospital) and list of countries where data will be collected. Reference to where list of study sites can be obtained	6
Eligibility criteria	#10	Inclusion and exclusion criteria for participants. If applicable, eligibility criteria for study centres and	6-7

For peer review only

		individuals who will perform the interventions (eg, surgeons, psychotherapists)	
Interventions: description	#11a	Interventions for each group with sufficient detail to allow replication, including how and when they will be administered	5
Interventions: modifications	#11b	Criteria for discontinuing or modifying allocated interventions for a given trial participant (eg, drug dose change in response to harms, participant request, or improving / worsening disease)	10-11
Interventions: adherance	#11c	Strategies to improve adherence to intervention protocols, and any procedures for monitoring adherence (eg, drug tablet return; laboratory tests)	10-11
Interventions: concomitant care	#11d	Relevant concomitant care and interventions that are permitted or prohibited during the trial	10-11
Outcomes	#12	Primary, secondary, and other outcomes, including the specific measurement variable (eg, systolic blood pressure), analysis metric (eg, change from baseline, final value, time to event), method of aggregation (eg, median, proportion), and time point for each outcome. Explanation of the clinical relevance of chosen efficacy and harm outcomes is strongly recommended	9
Participant timeline	#13	Time schedule of enrolment, interventions (including any run-ins and washouts), assessments, and visits for participants. A schematic diagram is highly recommended	Fig 1

For peer review only

		(see Figure)	
Sample size	#14	Estimated number of participants needed to achieve study objectives and how it was determined, including clinical and statistical assumptions supporting any sample size calculations	8
Recruitment	#15	Strategies for achieving adequate participant enrolment to reach target sample size	7 and 8
**Methods: Assignment of interventions (for controlled trials)**			
Allocation: sequence generation	#16a	Method of generating the allocation sequence (eg, computer-generated random numbers), and list of any factors for stratification. To reduce predictability of a random sequence, details of any planned restriction (eg, blocking) should be provided in a separate document that is unavailable to those who enrol participants or assign interventions	8-9
Allocation concealment mechanism	#16b	Mechanism of implementing the allocation sequence (eg, central telephone; sequentially numbered, opaque, sealed envelopes), describing any steps to conceal the sequence until interventions are assigned	8-9
Allocation: implementation	#16c	Who will generate the allocation sequence, who will enrol participants, and who will assign participants to interventions	8-9

For peer review only

Blinding (masking)	#17a	Who will be blinded after assignment to interventions (eg, trial participants, care providers, outcome assessors, data analysts), and how	14
Blinding (masking): emergency unblinding	#17b	If blinded, circumstances under which unblinding is permissible, and procedure for revealing a participant's allocated intervention during the trial	14
**Methods: Data collection, management, and analysis**			
Data collection plan	#18a	Plans for assessment and collection of outcome, baseline, and other trial data, including any related processes to promote data quality (eg, duplicate measurements, training of assessors) and a description of study instruments (eg, questionnaires, laboratory tests) along with their reliability and validity, if known. Reference to where data collection forms can be found, if not in the protocol	Table 1; 9, 11
Data collection plan: retention	#18b	Plans to promote participant retention and complete follow-up, including list of any outcome data to be collected for participants who discontinue or deviate from intervention protocols	10-11
Data management	#19	Plans for data entry, coding, security, and storage, including any related processes to promote data quality (eg, double data entry; range checks for data values).	15

For peer review only

		Reference to where details of data management procedures can be found, if not in the protocol	
Statistics: outcomes	#20a	Statistical methods for analysing primary and secondary outcomes. Reference to where other details of the statistical analysis plan can be found, if not in the protocol	14
Statistics: additional analyses	#20b	Methods for any additional analyses (eg, subgroup and adjusted analyses)	14
Statistics: analysis population and missing data	#20c	Definition of analysis population relating to protocol non-adherence (eg, as randomised analysis), and any statistical methods to handle missing data (eg, multiple imputation)	14
**Methods: Monitoring**			
Data monitoring: formal committee	#21a	Composition of data monitoring committee (DMC); summary of its role and reporting structure; statement of whether it is independent from the sponsor and competing interests; and reference to where further details about its charter can be found, if not in the protocol. Alternatively, an explanation of why a DMC is not needed	16
Data monitoring: interim analysis	#21b	Description of any interim analyses and stopping guidelines, including who will have access to these interim results and make the final decision to terminate the trial	14
Harms	#22	Plans for collecting, assessing, reporting, and managing solicited and spontaneously reported adverse events and other unintended effects of trial interventions or trial	15

For peer review only

		conduct	
Auditing	#23	Frequency and procedures for auditing trial conduct, if any, and whether the process will be independent from investigators and the sponsor	-
**Ethics and dissemination**			
Research ethics approval	#24	Plans for seeking research ethics committee / institutional review board (REC / IRB) approval	2, 18
Protocol amendments	#25	Plans for communicating important protocol modifications (eg, changes to eligibility criteria, outcomes, analyses) to relevant parties (eg, investigators, REC / IRBs, trial participants, trial registries, journals, regulators)	7
Consent or assent	#26a	Who will obtain informed consent or assent from potential trial participants or authorised surrogates, and how (see Item 32)	8
Consent or assent: ancillary studies	#26b	Additional consent provisions for collection and use of participant data and biological specimens in ancillary studies, if applicable	13
Confidentiality	#27	How personal information about potential and enrolled participants will be collected, shared, and maintained in order to protect confidentiality before, during, and after the trial	15
Declaration of	#28	Financial and other competing interests for principal	17

For peer review only

interests		investigators for the overall trial and each study site	
Data access	#29	Statement of who will have access to the final trial dataset, and disclosure of contractual agreements that limit such access for investigators	18
Ancillary and post trial care	#30	Provisions, if any, for ancillary and post-trial care, and for compensation to those who suffer harm from trial participation	-
Dissemination policy: trial results	#31a	Plans for investigators and sponsor to communicate trial results to participants, healthcare professionals, the public, and other relevant groups (eg, via publication, reporting in results databases, or other data sharing arrangements), including any publication restrictions	16
Dissemination policy: authorship	#31b	Authorship eligibility guidelines and any intended use of professional writers	-
Dissemination policy: reproducible research	#31c	Plans, if any, for granting public access to the full protocol, participant-level dataset, and statistical code	-
**Appendices**			
Informed consent materials	#32	Model consent form and other related documentation given to participants and authorised surrogates	-
Biological specimens	#33	Plans for collection, laboratory evaluation, and storage of biological specimens for genetic or molecular analysis in the current trial and for future use in ancillary studies, if applicable	-

For peer review only

None The SPIRIT checklist is distributed under the terms of the Creative Commons Attribution License CC-BY-ND 3.0. This checklist can be completed online using https://www.goodreports.org/, a tool made by the EQUATOR Network in collaboration with Penelope.ai

For peer review only

BMJ Open

Feasibility Study of Early Outpatient Review and Early Cardiac Rehabilitation After Cardiac Surgery: Mixed Methods Research Design – a study protocol

Journal:	*BMJ Open*
Manuscript ID	bmjopen-2019-035787.R1
Article Type:	Protocol
Date Submitted by the Author:	28-Nov-2019
Complete List of Authors:	Ngaage, Dumbor; Hull University Teaching Hospitals NHS Trust, Castle Hill Hospital<br>Mitchell, Natasha; University of York, York Trials Unit, Department of Health Sciences<br>Dean, Alexandra; University of York, York Trials unit, Department of Health Sciences<br>Hirst, Claire; University of York, York Trials Unit, Department of Health Sciences<br>Akowuah, Enoch; South Tees Hospitals NHS Foundation Trust, James Cook Hospital<br>Doherty, Patrick Joseph; University of York, Department of Health Sciences<br>Fairhurst, Caroline; University of York, York Trials unit, Department of Health Sciences<br>Flemming, Kate; University of York, Department of Health Sciences<br>Hewitt, Catherine; The University of York, York Trials Unit, Department of Health Sciences<br>Hinde, Sebastian; University of York, Centre for Health Economics<br>Mitchell, Alex; University of York, York Trials Unit, Department of Health Sciences<br>Nichols, Simon; Sheffield Hallam University - Collegiate Crescent Campus, Centre for Sport and Exercise Science<br>Watson, Judith; University of York, York Trials Unit, Department of Health Sciences
**Primary Subject Heading**:	Cardiovascular medicine
Secondary Subject Heading:	Surgery, Rehabilitation medicine, Nursing
Keywords:	REHABILITATION MEDICINE, Cardiothoracic surgery < SURGERY, STATISTICS & RESEARCH METHODS, HEALTH ECONOMICS

SCHOLARONE™
Manuscripts

Feasibility Study of Early Outpatient Review and Early Cardiac Rehabilitation After Cardiac Surgery: Mixed Methods Research Design – a study protocol

Authors:

Dumbor L Ngaage[1], Natasha Mitchell[2], Alexandra Dean[2], Claire Hirst[2], Enoch Akowuah[3], Patrick Doherty[2], Caroline Fairhurst[2], Kate Flemming[2], Catherine Hewitt[2], Sebastian Hinde[4], Alex Mitchell[2], Simon Nichols[5], Judith Watson[2]

Corresponding author: Mr Dumbor L Ngaage - Castle Hill Hospital, Hull University Teaching Hospitals NHS Trust, Castle Road Cottingham, Hull, UK; dumbor.ngaage@hey.nhs.uk; 01482623263

Author affiliations:

1. Castle Hill Hospital, Hull University Teaching Hospitals NHS Trust, Castle Road Cottingham, Hull, UK
   dumbor.ngaage@hey.nhs.uk - Orcid iD 0000-0003-2378-7160
2. Department of Health Sciences, University of York, ARRC Building, York, UK
   natasha.mitchell@york.ac.uk - Orcid iD 0000-0002-6904-2976
   alexandra.dean@york.ac.uk- Orcid iD 0000-0003-4711-5609
   claire.hirst@york.ac.uk - Orcid iD 0000-0001-7832-5030
   patrick.doherty@york.ac.uk Orcid iD 0000-0002-1887-0237
   caroline.fairhurst@york.ac.uk - Orcid iD 0000-0003-0547-462X
   kate.flemming@york.ac.uk - Orcid iD 0000-0002-0795-8516
   catherine.hewitt@york.ac.uk - Orcid iD 0000-0002-0415-3536
   alex.mitchell@york.ac.uk - Orcid iD 0000-0001-9311-2092
   jude.watson@york.ac.uk- Orcid iD 0000-0003-0694-3854
3. James Cook Hospital, South Tees Hospitals NHS Foundation Trust, Marton Road Middlesbrough, UK
   enoch.akowuah@nhs.net - Orcid iD 0000-0002-2429-3579
4. Centre for Health Economics, Alcuin A Block, University of York, York UK
   sebastian.hinde@york.ac.uk- Orcid iD 0000-0002-7117-4142
5. Centre for Sport and Exercise Science, Sheffield Hallam University, Collegiate Campus, Sheffield, UK
   S.J.Nichols@shu.ac.uk - Orcid iD 0000-0003-0377-6982

Main text word count: 3469 (excluding reference and tables)

For peer review only - http://bmjopen.bmj.com/site/about/guidelines.xhtml

Abstract

Introduction: Following cardiac surgery, patients currently attend an outpatient review six weeks after hospital discharge, where recovery is assessed and suitability to commence cardiac rehabilitation (CR) is determined. CR is then started from eight weeks. Following a median sternotomy, cardiac surgery patients are required to refrain from upper body exercises, lifting of heavy objects and other strenuous activities for 12 weeks. A delay in starting CR can prolong the recovery process, increase dependence on family/carers and can cause frustration. However, current guidelines for activity and exercise after median sternotomy have been described as restrictive, anecdotal and increasingly at odds with modern clinical guidance for CR. This study aims to examine the feasibility of bringing forward outpatient review and starting CR earlier.

Methods and analyses: This is a multi-centre, randomised controlled, open feasibility trial comparing postoperative outpatient review 6 weeks after hospital discharge, followed by CR commencement from 8 weeks (control arm) versus, postoperative outpatient review 3 weeks after hospital discharge, followed by commencement of CR from 4 weeks (intervention arm). The study aims to recruit 100 eligible patients, aged 18 to 80 years who have undergone elective or urgent cardiac surgery involving a full median sternotomy, over a 7 month period across two centres. Feasibility will be measured by consent, recruitment, retention rates, and attendance at appointments and CR sessions. Qualitative interviews with trial participants and staff will explore issues around study processes and acceptability of the intervention and the findings integrated with the feasibility trial outcomes to inform the design of a future full-scale randomised controlled trial (RCT).

Ethics and dissemination: Ethics approval was granted by East Midlands - Derby Research Ethics Committee on 10th January 2019. The findings will be presented at relevant conferences disseminated via peer-reviewed research publications, and to relevant stakeholders.

Trial registration: ISRCTN80441309

Keywords: cardiac surgery; cardiac rehabilitation; outpatient review; feasibility; median sternotomy

Protocol version: Version 2.0, 26th September 2019

For peer review only - http://bmjopen.bmj.com/site/about/guidelines.xhtml

ARTICLE SUMMARY

Strengths and limitations of this study

- This is one of the first studies to look at the timing of follow-up and cardiac rehabilitation after cardiac surgery.
- This feasibility study is a small multi-centre RCT.
- The study will collect both qualitative and quantitative data.
- If it is determined that a larger scale randomised controlled trial is feasible, this study will generate valuable data to enable its design.
- This feasibility study is not large enough to determine effectiveness or cost-effectiveness and is limited to assessing the feasibility of a larger trial.

For peer review only

INTRODUCTION

Following cardiac surgery, patients currently attend their first outpatient review six weeks after hospital discharge, where recovery is assessed and ability to commence cardiac rehabilitation (CR) is determined. CR is then started from eight weeks. In a 2017 survey, 35 of the 42 United Kingdom (UK) cardiac centres who responded, confirmed this as current practice [1]. This interval before review and CR extends the period of inactivity of patients, and medical attention for surgery-related complications is often sought during this period [2,3,4]. Our prospective observational study (FORCAST6) found that 39% of patients reported surgery-related complications in the six weeks following discharge, with the majority occurring in the first 4 weeks; 15% required hospital readmission. Although the majority of patients were satisfied with the six weeks interval, 44% felt it was too long and would have liked an earlier review [1].

The standard access for heart operations is through a median sternotomy [5,6,7,8]. In the UK, 35,158 heart operations were performed in 2015 [9]. Following a median sternotomy, patients are required to refrain from upper body exercises, lifting of heavy objects and other strenuous activities for 12 weeks [10,11,12,13]. These "sternal precautions" (SP) are intended to aid healing of the sternum. CR, which has significant short- and long-term benefits after cardiac surgery [14,15], is therefore delayed. The delay can mitigate the benefits of CR [16], contributing to physical deconditioning, and hinder the ability of CR to facilitate timely recovery of fitness and physical activity status [17]. However, current guidelines for activity and exercise after median sternotomy have been described as restrictive, conflicting, sometimes arbitrary, frequently anecdotal and frequently at odds with modern clinical guidance [11].

Following surgery, there are variations in types of exercises permitted, limits for weight of objects that can be lifted, guidelines for activities, and timeline for resumption of driving. Parker et al [18] demonstrated that the force elicited on the breastbone by coughing far exceeded lifting above the recommended limit. Adams and colleagues [11] investigated forces associated with 32 activities of daily living and reported that the majority not restricted by SP, such as opening and closing doors, generated forces greater than the allowed weight limit. While SP may help to support bone healing, the optimal nature and duration are unclear especially since sternal bone healing occurs by five weeks [12]. According to the 2013 UK

For peer review only - http://bmjopen.bmj.com/site/about/guidelines.xhtml

national audit of CR, late commencement contributes to a substantial number of heart surgery patients declining to participate [19]. Adverse outcomes following heart operations and other forms of surgery have been shown to be reduced by early patient review after hospital discharge [20,21]. The FORCAST6 study showed a high incidence of postoperative complications with the current 6-week patient review after heart surgery. This was highest in the first week after hospital discharge and declined to lowest levels by 4 weeks [1]. It therefore seems logical to conduct patient reviews after hospital discharge sooner than current practice to enable early CR from the period of postoperative stability and sternal bone healing at 5 weeks after surgery (4 weeks after hospital discharge).

The British Association for Cardiovascular Prevention and Rehabilitation (BACPR) Standards and Core Components [22] and the National Certification Programme for CR [23] recommend early commencement of CR. This is monitored by the National Audit of CR 2018 [19]. A delay in starting CR can prolong the recovery process, increase dependence on family/carers and can cause frustration. This may contribute to anxiety and depression that is reported in patients recovering after cardiac surgery [24].

This study aims to examine the feasibility of bringing forward outpatient review and CR in order to facilitate recovery, physical fitness and quality of life.

METHODS AND ANALYSIS

Study design

A multi-centre, randomised controlled, open feasibility trial using mixed methods to establish the feasibility of conducting a study where:

– Participants have a postoperative outpatient review 6 weeks after hospital discharge, followed by commencement of CR from 8 weeks (*control arm*); or

– Participants have a postoperative outpatient review 3 weeks after hospital discharge, followed by commencement of CR from 4 weeks (*intervention arm*).

For peer review only - http://bmjopen.bmj.com/site/about/guidelines.xhtml

The study is detailed in Figure 1: Study Flowchart and the protocol follows the SPIRIT reporting guidelines [25].

Study aim

To establish the feasibility of delivering and evaluating a definitive randomised controlled trial (RCT) of the current 8th week versus 4th week CR commencement pathways, reducing the period over which patients are required to refrain from usual physical activities following cardiac surgery.

Study objectives

The primary objective is to determine the feasibility of delivering outpatient review three weeks after discharge post-cardiac surgery, followed by CR from four weeks.

Secondary objectives

- Assess surgeons' and surgical practitioners' willingness to conduct outpatient review three weeks after discharge and refer patients to CR.
- Examine barriers to patient enrolment.
- Identify recruitment rates and drop-out to follow-up.
- Identify the most appropriate outcome measures.
- Test follow-up procedures and data collection tools and management.
- Assess the feasibility of conducting an economic evaluation of any future definitive RCT.
- Gather outcome data for power and sample size calculations for the RCT.
- Inform any necessary redesign of a new recovery pathway in light of information gained.

Study population and setting

Patients attending for cardiac surgery at Hull University Teaching Hospitals National Health Service (NHS) Trust, UK and South Tees Hospitals NHS Foundation Trust, UK will be approached regarding study participation.

For peer review only - http://bmjopen.bmj.com/site/about/guidelines.xhtml

Inclusion criteria

- Undergone elective or urgent cardiac surgery, including the following procedures (isolated or combined):
    - Coronary artery bypass graft (CABG)
    - Aortic valve replacement
    - Mitral valve repair or replacement
    - Atrial fibrillation ablation
    - Left atrial appendage occlusion
- Has had a full median sternotomy
- Willing and capable of giving informed consent
- Aged 18 to 80 years at the time of giving consent
- Able to self-complete the English language outcome measure tools (or can complete with assistance)
- Able to follow detailed verbal instructions required for clinical assessments.

Exclusion criteria

- Body mass index (BMI) >45kg/m^2
- Heart failure with left ventricular ejection fraction of <30% before surgery
- Early postoperative sternal wound complications (e.g. deep sternal wound infection, wound dehiscence and/or sternal instability)
- Postoperative complications resulting in prolonged hospital stay >14 days after surgery.

Amendment to eligibility criteria

During the initial recruitment period, fewer patients than anticipated were meeting the eligibility criteria, and many were 'out of area' impacting on their ability to attend study CR locations. With the funder's approval, a substantial amendment was submitted to the ethics committee. The original criteria had only included patients who had undergone CABG, had an

For peer review only - http://bmjopen.bmj.com/site/about/guidelines.xhtml

upper age limit of 75 years and a maximum BMI limit of 40kg/m^2. This amendment was approved on 30th October 2019.

Sample size

This study is a feasibility RCT and therefore does not have a primary outcome measure to inform a power calculation. Sample sizes of between 24 and 70 have been recommended for feasibility trials to allow for the reliable estimation of a standard deviation for use in future sample size calculations [26,27]. The plan is to recruit 100 eligible patients, allowing for a 30% attrition rate in order to still have 70 patients in the final analysis. There will be a 7 month recruitment period across two centres.

Participant recruitment

Patients scheduled for elective cardiac surgery will be given the study leaflet prior to surgery. Patients undergoing urgent cardiac surgery will receive it following surgery. All patients will undergo surgery as normal, receiving standard in-hospital postoperative care.

Eligibility and consent

After discharge from intensive care to the ward following surgery, the medical staff will review patients' routine clinical examination results, including sternal stability and postoperative tests. Those identified as potentially eligible, interested and willing, will have their eligibility confirmed by the Research Nurse using an eligibility checklist and written informed consent will be obtained according to Good Clinical Practice guidelines. Consent will also be sought to contact participants who are interested in being interviewed as part of an embedded qualitative study.

Patients screened as eligible, but who develop complications between being considered ready for discharge and going home, will not proceed to consent until deemed well enough (if remain in hospital for >14 days after surgery, as per exclusion criteria they will not proceed at all).

For peer review only - http://bmjopen.bmj.com/site/about/guidelines.xhtml

Randomisation

Following surgery, consent, and completion of baseline data collection and assessments, participants will be randomised to either the control or intervention arm.

Randomisation will be performed by a remote, centralised randomisation service provided by York Trials Unit (YTU), and the allocation sequence generated by a statistician not involved in the study. Participants will be individually randomised and stratified according to study site on a 1:1 basis using variable block sizes. Authorised site research staff will telephone the randomisation service to obtain the participant's allocation.

Outcomes

Participant self-reported data will be collected at baseline, prior to CR commencement, following CR completion and at final follow-up assessment (up to 26 weeks post-randomisation):

i. Demographics (baseline only)
ii. EQ-5D-5L: a validated generic patient-reported outcome measure. [28]
iii. NHS resource use.

The following clinical data will be collected:

i) height, weight, BMI, pre-operative presentation (baseline only);
ii) heart rate, blood pressure, oxygen saturation at baseline, outpatient review and pre-CR as per usual practice;
iii) physical fitness assessed by dynamic testing with incremental shuttle walk test (ISWT) for both study arms at commencement and end of CR, and at final follow-up;
iv) cardiopulmonary fitness assessed by cardiopulmonary exercise testing at baseline and at final follow-up, for up to 25 patients in each study arm [Hull study site only];
v) 30- and 90-day mortality, surgical site complications and hospital readmission rates.

In line with the objectives of a feasibility study, we will also gather information on:

vi) recruitment rates and drop-out to follow-up;
vii) compliance to treatment arm allocation;

For peer review only - http://bmjopen.bmj.com/site/about/guidelines.xhtml

viii) acceptability of patient recruitment, early outpatient review and CR to patients, clinicians and NHS organisations.

Cardiopulmonary exercise testing (CPET)

Maximal CPET provides a holistic assessment of the cardiovascular, ventilatory and metabolic responses to exercise and is a powerful diagnostic and prognostic tool [29]. Maximal CPET is the gold-standard method of assessing changes in aerobic fitness which is potentially important if we are to assess the beneficial/not harmful effects of early CR. Maximal CPET will be conducted on a cycle ergometer with patients pedalling at 50 revolutions per minute throughout the test. The test workload will start at 0watts and increase in 10watts/minute increments until the patient reaches volitional exhaustion (ideally 8-12 minutes [29]). Maximal CPET will only be carried out (after randomisation) in up to 25 participants in each arm in the Hull site only due to costs and logistics, but also to ensure that test procedures and data interpretation are consistent. CPET will be completed once a participant is deemed clinically stable and conducted within 14 days after surgery. It will be repeated at the final follow-up.

Postoperative outpatient review

As standard practice, all participants will have a postoperative outpatient clinical review in order to be certified suitable to commence CR. This clinical decision is usually based on the absence of limiting complications. This review will take place at six weeks post-hospital discharge in the control arm, and three weeks post-discharge in the intervention arm. Participants considered unfit for CR will be given a second review appointment for approximately one week later and if necessary, a third review appointment two weeks later. Reason(s) they were deemed unfit will be documented. If they are deemed unfit for CR at the third review, they will not commence CR as part of the FARSTER study. They will, however, continue to be monitored as per usual practice and be sent all study questionnaires for completion.

Pre-cardiac rehabilitation assessment

Referred participants will be offered a comprehensive programme and an assessment prior to starting their programme. Participants will receive a holistic assessment from a specialist

For peer review only - http://bmjopen.bmj.com/site/about/guidelines.xhtml

physiotherapist or exercise professional including exercise testing using the ISWT to help personalise their exercise prescription. The ISWT is a popular aerobic fitness assessment tool in UK CR programmes and recommended by the CR physiotherapist association [30, 31]. Although reference values for ISWT are available in conventional CR patient populations [32], they have not yet been validated for use in the early CR cohorts. If a participant misses this appointment they will be sent another.

Cardiac rehabilitation programme

This will consist of supervised low-to-moderate intensity exercise performed once (South Tees) or twice (Hull) a week for eight weeks (as is standard UK practice). Exercise will be prescribed according to standards published by BACPR [22]. Attendance at these sessions and session content will be recorded for each participant.

Post-cardiac rehabilitation appointment

Following completion of the CR programme (16 weeks post-discharge for the control arm and 12 weeks post-discharge for the intervention arm), all participants will have a repeat ISWT. If a participant misses this appointment they will be sent another. A discharge letter will be sent to participants' General Practitioners summarising their treatment.

Final follow up assessment

At final follow-up (up to 26 weeks after randomisation), participants will have a repeat CPET (if appropriate), undergo a final ISWT, have an end-of-study consultation and be examined for sternal wound complications, including sternal instability. Medical history will be taken to establish hospital readmissions and Accident & Emergency department attendance for surgery-related complications. If a participant misses this appointment they will be sent another.

DATA COLLECTION

At pre-CR and post-CR appointments and the final follow up assessment, participants will be asked to complete quality of life (EQ-5D-5L) and resource use questions (see Table 1: Study Assessment Schedule). If a participant does not attend, the questionnaire will be posted out

For peer review only - http://bmjopen.bmj.com/site/about/guidelines.xhtml

to them, with two reminders subsequently sent if no response (one and two weeks later as necessary).

For peer review only

Table 1: Study assessment schedule

			Control arm					Intervention arm				
**Procedure**	**Surgery**	**Eligibility/ Discharge**	**Outpatient appointment**	**Pre-CR**	**CR sessions** (8-16 sessions over 8 weeks)	**Post-CR**	**Final follow up visit**	**Outpatient appointment**	**Pre-CR**	**CR sessions** (8-16 sessions over 8 weeks)	**Post-CR**	**Final follow up visit**
	Week -1	*Week 0*	*Week 6#*	*Week 8*		*Week 16*	*Week 26*	*Week 3*	*Week 4*		*Week 12*	*Week 26*
Surgery	●*											
Screening for eligibility		●										
Consent		●										
Baseline data collection		●										
Randomisation		●										
Clinical examination		●*	●*				●	●*				●
Cardiopulmonary exercise test		●^					●^					●^
Incremental shuttle walk test				●		●	●		●		●	●
EQ-5D-5L		●		●		●	●		●		●	●
Cardiac rehabilitation				●*		●*			●*		●*	
Vital signs – heart rate, blood pressure, oxygen saturation		●*	●*	●*				●*	●*			
Participant interviews+			●				●	●				●
Session attendance					●					●		
Session content					●					●		
Participant diary					●				●		●	
Resource use questions				●		●	●		●		●	●
Adverse event monitoring			●*	●		●	●	●*	●		●	●

* part of normal patient pathway; ^ for only 25 participants in each group from Hull site; + for a purposive sample of up to 10 patients in each treatment arm

For peer review only

For peer review only - http://bmjopen.bmj.com/site/about/guidelines.xhtml

QUALITATIVE DATA COLLECTION

After the outpatient review, we will conduct semi-structured interviews by telephone with approximately ten participants from each treatment arm (20 in total) to determine their views on the timing of their postoperative outpatient review and readiness to commence CR. These participants will be purposively selected from those who agreed to be approached for interview. A second interview will be held with the same participants on their completion of the study to determine their views on the timing of the outpatient review and CR, study conduct and processes. All interviews will be digitally recorded with participant permission.

Participants will be encouraged to complete bespoke CR diaries for the eight weeks of CR detailing their experience at each session, including willingness to take part and any difficulties.

Research Nurses will complete a diary detailing recruitment of patients, reasons for non-consent, and any difficulties encountered with patient follow-up. CR staff will be asked to keep a diary collecting information about participant drop-outs/opt-outs, level of participation and adverse events during the sessions. In addition, staff involved in the research will be invited a focus group at the end of final follow-up to discuss any difficulties encountered with recruitment, outpatient review, CR sessions and follow-up. Organisational and clinical barriers will also be examined.

Data will be analysed separately for patients, research staff and clinical staff using conventional content analysis [33]. Transcribed data from the interviews and focus groups will be downloaded into qualitative data analysis computer software package, coded and analysed inductively using thematic content analysis [34] to inform the development of the logistical processes involved in delivering a large-scale multi-centre RCT.

STATISTICAL ANALYSES

For peer review only

A full statistical analysis plan detailing intended analyses will be drafted before completion of data collection. Analysis and reporting will follow CONSORT guidelines [35,36] and a flow diagram will depict the progression of participants through the trial. Baseline data will be summarised by randomised group with no formal statistical comparisons undertaken. Continuous variables will be summarised using descriptive statistics (n, mean, standard deviation, median, minimum and maximum) while categorical data will be reported as counts and percentages. Participant outcomes will be summarised descriptively by randomised group and time point, including extent of missing data. As this is a feasibility trial, formal hypothesis testing for effectiveness will not be carried out. The number of participants attending their review appointment and entering CR, and the time between surgery and these events, will be summarised by treatment group. Questionnaire return rates will be presented and adverse events will be summarised descriptively. No interim analyses will be conducted.

HEALTH ECONOMICS ANALYSIS

A full cost-effectiveness analysis will not be undertaken as part of this feasibility study. Instead, the study will consider the feasibility of collecting the data needed for an economic analysis of a full scale trial and explore the rate of response and missingness of relevant questionnaires. Estimates of patient benefit, determined from the EQ-5D-5L, and NHS resource use, using patient reported questionnaires, will be summarised for the two arms of the trial. Mortality and adverse event rates will also be monitored to determine the importance of long term extrapolation of related outcomes for any future analysis of a full scale RCT. As this is a feasibility trial, formal estimation of the cost-effectiveness of the respective interventions will not be carried out.

BLINDING

By the nature of the timing of the study treatments, blinding of the participants and clinicians is not possible and a procedure for unblinding is not necessary. The clinician evaluating the CPET data will be blinded. The statistician and health economist will be blinded to study allocation.

For peer review only - http://bmjopen.bmj.com/site/about/guidelines.xhtml

SAFETY MEASUREMENTS

Adverse events (AEs) and serious adverse events (SAE), will be recorded throughout the study. Intensity and relationship to the study intervention will be described. Examples of AE related to participation in this study would be: atrial fibrillation; sternal pain, and; undue fatigue or exhaustion. An example of a SAE would be sternal instability.

Participants will undergo clinical assessment after discharge and be certified suitable for CR by the surgical team. Before commencing CR they will undergo exercise testing, and CR will be tailored to their fitness levels. At completion of CR, they will have another assessment and exit consultation.

Information about AEs whether patient reported, discovered via clinical team questioning, identified through physical examination or in medical notes, will be recorded up to and including final follow-up. Ongoing review of AEs will take place during monthly Trial Management Group (TMG) meetings, discussed with the Patient Advisory Group (PAG) and Trial Steering Committee (TSC) and reported to the sponsor and Research Ethics Committee in line with their guidelines.

DATA COLLECTION, INTEGRITY AND MANAGEMENT

Data collected as part of this research includes questionnaires, clinical assessments, information from medical records and qualitative data from interviews. Data will be collected through designed questionnaires identified by a unique participant trial number only. Each site will hold data according to the current Data Protection Act (2018) [37] and the General Data Protection Regulation (May 2018) [38].

All information collected during the study will be kept strictly confidential and stored on a secure password-protected server located at the University of York, for the purposes of assisting in follow-ups during the study. All paper documents will be stored securely. Electronic sound files and transcripts from qualitative interviews will be assigned a unique participant number, known only to the qualitative researcher and appropriate members of

For peer review only - http://bmjopen.bmj.com/site/about/guidelines.xhtml

the research team. Any quotes published will be anonymous. All data will be archived for ten years following the end of the study.

PATIENT AND PUBLIC INVOLVEMENT

A PAG will hold meetings with research nurses, CR staff and sites' Principal investigators during the study to discuss any challenges encountered and how to overcome them. The group act as an important source of reference and the research progress will be discussed. Their role initially included assisting with the development of study documentation ensuring material was clear to the lay public; identifying barriers to participation and possible mitigation of those barriers; and providing input for topic guides for the qualitative research. Towards completion of the study, the PAG will identify appropriate pathways for dissemination and contribute to writing the lay summary.

ETHICS, OVERSIGHT AND DISSEMINATION

The TMG, led by the Chief Investigator, are responsible for overseeing the day-to-day running and management of the trial with YTU responsible for project management. Due to the low risk nature of this trial, one independent steering and monitoring committee will fulfil the roles traditionally undertaken by the TSC and Data Monitoring Committee. It will comprise of a Chair, statistician, one other independent member, and Public Involvement representative.

Study findings will be discussed with participants, PAG, and at a meeting of local General Practitioners. Study results will be presented at annual conferences of The Society for Cardiothoracic Surgery of Great Britain and Ireland, BACPR, and The European Association for Cardiothoracic Surgery. Study results will also be submitted to the funders and peer-reviewed journals.

Figure legend

Figure 1: FARSTER study flowchart

For peer review only

Acknowledgements

The authors would like to thank Carmen Neave and Karen Dobbs, the Research Nurses at the sites; all the clinical and CR staff at the participating sites; and the members of the PAG.

Author Contributions

DN, PD and SN led on the conception, design and writing of the study. DN led the grant application and as Chief Investigator, takes overall responsibility for the trial. JW led on the writing of the study protocol with substantial contributions to the design, writing, critical review of intellectual content and final manuscript approval from DN, NM, AD, CHi, EA, PD, CF, KF, CHe, SB, AM and SN. CF, CHe and AM provided statistical expertise in the study design and development stages of the project and the protocol. CHi, AD, NM and JW made substantial contributions to the trial design and management. KF led on the qualitative study design. SH was specifically responsible for the health economic aspects of the study design and DN, EA, PD and SN were responsible for providing expert clinical support. All authors agree to be accountable for their work.

Funding statement

Hull University Teaching Hospitals NHS Trust are the sponsors on behalf of the funder, who is the NIHR as stated below. This paper presents independent research funded by the National Institute for Health Research (NIHR) under its Research for Patient Benefit (RfPB) Programme (Grant Reference Number PB-PG-0317-20047).

Disclaimer

The views expressed are those of the author(s) and not necessarily those of the NHS, the NIHR, or the Department of Health and Social Care.

Competing interests

None declared.

Patient consent for publication

Not required.

Ethics approval

Ethics approval was granted by East Midlands - Derby Research Ethics Committee on 10th January 2019 (REC Reference: 18/EM/0391).

Data availability statement

There are no data in this work, as this is a trial protocol.

For peer review only - http://bmjopen.bmj.com/site/about/guidelines.xhtml

REFERENCES

1. Ngaage DL, Gooseman M, Bulliment K, et al. Is six weeks too long for first planned postoperative review after cardiac surgery? FORCAST6. *Br J Cardiol* 2019;26:34 doi:10.5837/bjc.2019.008
2. Shroyer AL, Coombs LP, Peterson ED, et al. The Society of Thoracic Surgeons: 30-day operative mortality and morbidity risk models. *Ann Thor Surg* 2003; 75(6): 1856-64; discussion 1864-5.
3. Fox JP, Suter LG, Wang K, et al. Hospital-based, acute care use among patients within 30 days of discharge after coronary artery bypass surgery. *Ann Thorac Surg* 2013; 96(1):96-104
4. Hannan EL, Zhong Y, Lahey SJ, et al. 30-day readmissions after coronary artery bypass graft surgery in New York State. *JACC Cardiovasc Interv*. 2011; May;4(5):569-76.
5. Braimbridge MV. Median Sternotomy. *Lancet.* 1965; 1(7385):585.
6. Fischer H. [Evaluation of medial sternotomy as an approach to the heart]. *Zentralbl Chir* 1956; 81(40):2099-101.
7. Gerbode F, Braimbridge MV, Melrose DG. Median sternotomy for open cardiac surgery during total heart-lung by-pass. *AMA Arch Surg* 1958;76(5):821-3; discussion 823-4.
8. Lillehei CW, Cardozo RH. Use of median sternotomy with femoral artery cannulation in open cardiac surgery. *Surg Gynecol Obstet* 1959;108(6):706-14.
9. The Society for Cardiothoracic Surgery in Great Britain & Ireland. National Adult Cardiac Database Report. Available at http://bluebook.scts.org/. Accessed June 05, 2017.
10. British Heart Foundation. Cardiac rehabilitation. Available at www.bhf.org.uk/publications. Accessed November 26, 2017.
11. Adams J, Cline MJ, Hubbard M, et al. A new paradigm for post-cardiac event resistance exercise guidelines. *Am J Cardiol* 2006; 97(2):281-6.
12. Brocki BC, Thorup CB, Andreasen JJ. Precautions related to midline sternotomy in cardiac surgery: a review of mechanical stress factors leading to sternal complications. *Eur J Cardiovasc Nurs* 2010; 9(2):77-84.
13. Cahalin LP, Lapier TK, Shaw DK. Sternal Precautions: Is It Time for Change? Precautions versus Restrictions - A Review of Literature and Recommendations for Revision. *Cardiopulm Phys Ther* J 2011; 22(1):5-15.
14. Anderson L, Thompson DR, Oldridge N, et al. Exercise-based cardiac rehabilitation for coronary heart disease (Review). *Cochrane Database of Systematic Reviews* 2016, Issue 1. Art. No.: CD001800. DOI: 10.1002/14651858.CD001800.pub3.
15. Pack QR, Goel K, Lahr BD, et al. Participation in cardiac rehabilitation and survival after coronary artery bypass graft surgery a community-based study. *Circulation*. 2013; 128:590-7.
16. Marzolini S, Blanchard C, Alter DA, et al. Delays in Referral and Enrolment Are Associated With Mitigated Benefits of Cardiac Rehabilitation After Coronary Artery Bypass Surgery. *Circ Cardiovasc Qual Outcomes.* 2015; Nov;8(6):608-20

For peer review only - http://bmjopen.bmj.com/site/about/guidelines.xhtml

17. LaPier TK. Functional status of patients during subacute recovery from coronary artery bypass surgery. *Heart Lung* 2007; 36(2):114-24
18. Parker R, Adams JL, Ogola G, et al. Current activity guidelines for CABG patients are too restrictive: comparison of the forces exerted on the median sternotomy during a cough vs. lifting activities combined with valsalva maneuver. *Thorac Cardiovasc Surg* 2008;56(4):190-4.
19. NACR 2018. British Heart Foundation National Audit of Cardiac Rehabilitation. Quality and Outcomes Report. https://www.bhf.org.uk/informationsupport/publications/statistics/national-audit-of-cardiac-rehabilitation-quality-and-outcomes-report-2018
20. Hall MH, Esposito RA, Pekmezaris R, et al. Cardiac surgery nurse practitioner home visits prevent coronary artery bypass graft readmissions. *Ann Thorac Surg*. 2014; May;97(5):1488-93; discussion 1493-5
21. Saunders RS, Fernandes-Taylor S, Rathouz PJ, et al. Outpatient follow-up versus 30-day readmission among general and vascular surgery patients: a case for redesigning transitional care. *Surgery*. 2014; Oct;156(4):949-56.
22. BACPR. Standards and Core Components for Cardiovascular Disease Prevention and Rehabilitation. 2017. Available from: http://www.bacpr.com
23. http://www.cardiacrehabilitation.org.uk/NCP-CR.htm
24. Tully PJ, Baker RA. Depression, anxiety, and cardiac morbidity outcomes after coronary artery bypass surgery: a contemporary and practical review**.** *J Geriatric* Cardiol 2012; 9: 197–208.
25. Chan A-W, Tetzlaff JM, Altman DG, Laupacis A, Gøtzsche PC, Krleža-Jerić K, Hróbjartsson A, Mann H, Dickersin K, Berlin J, Doré C, Parulekar W, Summerskill W, Groves T, Schulz K, Sox H, Rockhold FW, Rennie D, Moher D. SPIRIT 2013 Statement: Defining standard protocol items for clinical trials. Ann Intern Med. 2013;158(3):200-207
26. Julious SA, Sample size of 12 per group rule of thumb for a pilot study. *Pharmaceutical Statistics*, 2005; **4**(4): p. 287-291
27. Teare MD, et al., Sample size requirements to estimate key design parameters from external pilot randomised controlled trials: a simulation study. *Trials,* 2014; **15**(1): p. 264.
28. Hurst NP, Kind P, Ruta D, et al. Measuring health-related quality of life in rheumatoid arthritis: Validity, responsiveness and reliability of EuroQol (EQ-5D). *Br J Rheumatol.* 1997; 36 (5):551-9.
29. Taylor C, Nichols S, Ingle L (2015) A clinician's guide to cardiopulmonary exercise testing 1: an introduction. *Br J Hosp Med* 76(4): 192–5 (doi: 10.12968/hmed.2015.76.4.192)
30. Grove T. Incremental shuttle walk test in cardiac rehabilitation. *B J Card Nurs,* 2013; 8:1, 31-37
31. ACPICR. Standards for physical activity and exercise in the cardiovascular population. 3rd ed. Heather P, Helen B, Samantha B, John B, Laura Burgess, Keri G, et al., editors: Association of Chartered Physiotherapists in Cardiac Rehabilitation 2015.

For peer review only - http://bmjopen.bmj.com/site/about/guidelines.xhtml

32. Doherty P, Alotaibi JFM. Evaluation of determinants of walking fitness in patients attending cardiac rehabilitation. *BMJ Open Sport Exerc Med* 2017;2:e000203. doi:10.1136/bmjsem-201600020
33. Hsieh, H-F, Shannon SE. Three Approaches to Qualitative Content Analysis. *Qualitative Health Research*. 2005; **15**(9): 1277-1288
34. Green J, Thorogood N. Qualitative Methods for Health Research 4th Edition. London, Sage. 2018.
35. Schulz KF, Altman DG, MoherD, CONSORT 2010 statement: updated guidelines for reporting parallel group randomized trials. *Ann Intern Med,* 2010; **152**(11): p. 726-32.
36. Eldridge SM, Chan CL, Campbell MJ et al., CONSORT 2010 statement: extension to randomised pilot and feasibility trials. BMJ, 2016; **355**.
37. Data Protection Act, UK Government http://www.legislation.gov.uk/ukpga/2018/12/contents/enacted
38. Information Commissioner's Office https://ico.org.uk/for-organisations/guide-to-the-general-data-protection-regulation-gdpr/

For peer review only

For peer review only - http://bmjopen.bmj.com/site/about/guidelines.xhtml

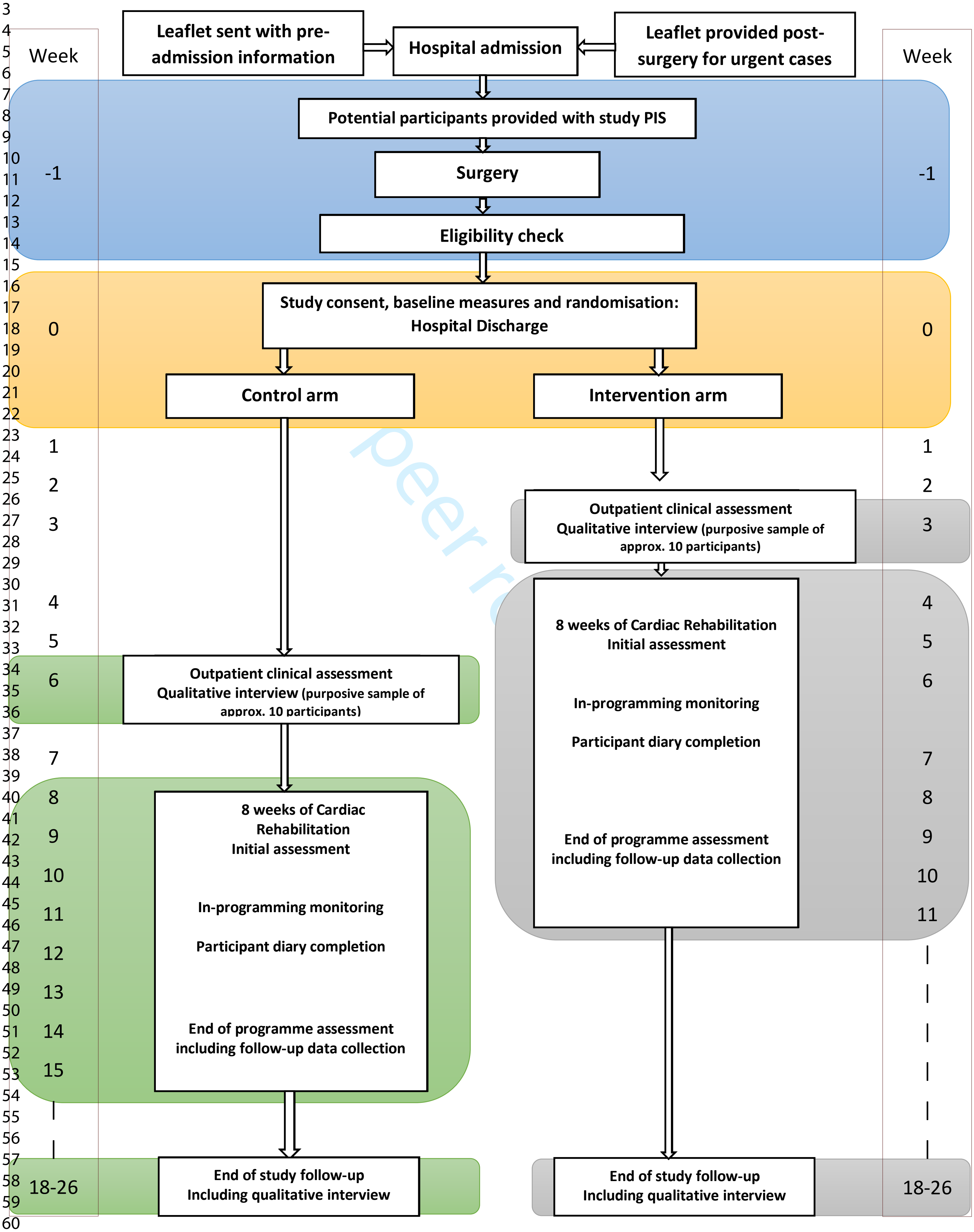
Week
Leaflet sent with pre-admission information
Hospital admission
Leaflet provided post-surgery for urgent cases
Week
-1
Potential participants provided with study PIS
Surgery
Eligibility check
-1
0
Study consent, baseline measures and randomisation: Hospital Discharge
0
Control arm
Intervention arm
1
2
3
4
5
6
7
8
9
10
11
12
13
14
15
18-26
Outpatient clinical assessment
Qualitative interview (purposive sample of approx. 10 participants)
8 weeks of Cardiac Rehabilitation
Initial assessment
In-programming monitoring
Participant diary completion
End of programme assessment including follow-up data collection
End of study follow-up
Including qualitative interview
Outpatient clinical assessment
Qualitative interview (purposive sample of approx. 10 participants)
8 weeks of Cardiac Rehabilitation
Initial assessment
In-programming monitoring
Participant diary completion
End of programme assessment including follow-up data collection
End of study follow-up
Including qualitative interview
1
2
3
4
5
6
7
8
9
10
11
18-26

Reporting checklist for protocol of a clinical trial.

Based on the SPIRIT guidelines.

Instructions to authors

Complete this checklist by entering the page numbers from your manuscript where readers will find each of the items listed below.

Your article may not currently address all the items on the checklist. Please modify your text to include the missing information. If you are certain that an item does not apply, please write "n/a" and provide a short explanation.

Upload your completed checklist as an extra file when you submit to a journal.

In your methods section, say that you used the SPIRITreporting guidelines, and cite them as:

Chan A-W, Tetzlaff JM, Altman DG, Laupacis A, Gøtzsche PC, Krleža-Jerić K, Hróbjartsson A, Mann H, Dickersin K, Berlin J, Doré C, Parulekar W, Summerskill W, Groves T, Schulz K, Sox H, Rockhold FW, Rennie D, Moher D. SPIRIT 2013 Statement: Defining standard protocol items for clinical trials. Ann Intern Med. 2013;158(3):200-207

		Reporting Item	Page Number
**Administrative information**			
Title	#1	Descriptive title identifying the study design, population, interventions, and, if applicable, trial acronym	1

For peer review only

Trial registration	#2a	Trial identifier and registry name. If not yet registered, name of intended registry	2
Trial registration: data set	#2b	All items from the World Health Organization Trial Registration Data Set	-
Protocol version	#3	Date and version identifier	2
Funding	#4	Sources and types of financial, material, and other support	17
Roles and responsibilities: contributorship	#5a	Names, affiliations, and roles of protocol contributors	1, 17
Roles and responsibilities: sponsor contact information	#5b	Name and contact information for the trial sponsor	17
Roles and responsibilities: sponsor and funder	#5c	Role of study sponsor and funders, if any, in study design; collection, management, analysis, and interpretation of data; writing of the report; and the decision to submit the report for publication, including whether they will have ultimate authority over any of these activities	17
Roles and responsibilities: committees	#5d	Composition, roles, and responsibilities of the coordinating centre, steering committee, endpoint adjudication committee, data management team, and other individuals or groups overseeing the trial, if applicable (see Item 21a for data monitoring committee)	16

For peer review only

**Introduction**			
Background and rationale	#6a	Description of research question and justification for undertaking the trial, including summary of relevant studies (published and unpublished) examining benefits and harms for each intervention	4-5
Background and rationale: choice of comparators	#6b	Explanation for choice of comparators	4-5
Objectives	#7	Specific objectives or hypotheses	6
Trial design	#8	Description of trial design including type of trial (eg, parallel group, crossover, factorial, single group), allocation ratio, and framework (eg, superiority, equivalence, non-inferiority, exploratory)	5
**Methods: Participants, interventions, and outcomes**			
Study setting	#9	Description of study settings (eg, community clinic, academic hospital) and list of countries where data will be collected. Reference to where list of study sites can be obtained	6
Eligibility criteria	#10	Inclusion and exclusion criteria for participants. If applicable, eligibility criteria for study centres and	6-7

For peer review only

		individuals who will perform the interventions (eg, surgeons, psychotherapists)	
Interventions: description	#11a	Interventions for each group with sufficient detail to allow replication, including how and when they will be administered	5
Interventions: modifications	#11b	Criteria for discontinuing or modifying allocated interventions for a given trial participant (eg, drug dose change in response to harms, participant request, or improving / worsening disease)	10-11
Interventions: adherance	#11c	Strategies to improve adherence to intervention protocols, and any procedures for monitoring adherence (eg, drug tablet return; laboratory tests)	10-11
Interventions: concomitant care	#11d	Relevant concomitant care and interventions that are permitted or prohibited during the trial	10-11
Outcomes	#12	Primary, secondary, and other outcomes, including the specific measurement variable (eg, systolic blood pressure), analysis metric (eg, change from baseline, final value, time to event), method of aggregation (eg, median, proportion), and time point for each outcome. Explanation of the clinical relevance of chosen efficacy and harm outcomes is strongly recommended	9
Participant timeline	#13	Time schedule of enrolment, interventions (including any run-ins and washouts), assessments, and visits for participants. A schematic diagram is highly recommended	Fig 1

For peer review only

		(see Figure)	
Sample size	#14	Estimated number of participants needed to achieve study objectives and how it was determined, including clinical and statistical assumptions supporting any sample size calculations	8
Recruitment	#15	Strategies for achieving adequate participant enrolment to reach target sample size	7 and 8
**Methods: Assignment of interventions (for controlled trials)**			
Allocation: sequence generation	#16a	Method of generating the allocation sequence (eg, computer-generated random numbers), and list of any factors for stratification. To reduce predictability of a random sequence, details of any planned restriction (eg, blocking) should be provided in a separate document that is unavailable to those who enrol participants or assign interventions	8-9
Allocation concealment mechanism	#16b	Mechanism of implementing the allocation sequence (eg, central telephone; sequentially numbered, opaque, sealed envelopes), describing any steps to conceal the sequence until interventions are assigned	8-9
Allocation: implementation	#16c	Who will generate the allocation sequence, who will enrol participants, and who will assign participants to interventions	8-9

For peer review only

Blinding (masking)	#17a	Who will be blinded after assignment to interventions (eg, trial participants, care providers, outcome assessors, data analysts), and how	14
Blinding (masking): emergency unblinding	#17b	If blinded, circumstances under which unblinding is permissible, and procedure for revealing a participant's allocated intervention during the trial	14
**Methods: Data collection, management, and analysis**			
Data collection plan	#18a	Plans for assessment and collection of outcome, baseline, and other trial data, including any related processes to promote data quality (eg, duplicate measurements, training of assessors) and a description of study instruments (eg, questionnaires, laboratory tests) along with their reliability and validity, if known. Reference to where data collection forms can be found, if not in the protocol	Table 1; 9, 11
Data collection plan: retention	#18b	Plans to promote participant retention and complete follow-up, including list of any outcome data to be collected for participants who discontinue or deviate from intervention protocols	10-11
Data management	#19	Plans for data entry, coding, security, and storage, including any related processes to promote data quality (eg, double data entry; range checks for data values).	15

For peer review only

		Reference to where details of data management procedures can be found, if not in the protocol	
Statistics: outcomes	#20a	Statistical methods for analysing primary and secondary outcomes. Reference to where other details of the statistical analysis plan can be found, if not in the protocol	14
Statistics: additional analyses	#20b	Methods for any additional analyses (eg, subgroup and adjusted analyses)	14
Statistics: analysis population and missing data	#20c	Definition of analysis population relating to protocol non-adherence (eg, as randomised analysis), and any statistical methods to handle missing data (eg, multiple imputation)	14
**Methods: Monitoring**			
Data monitoring: formal committee	#21a	Composition of data monitoring committee (DMC); summary of its role and reporting structure; statement of whether it is independent from the sponsor and competing interests; and reference to where further details about its charter can be found, if not in the protocol. Alternatively, an explanation of why a DMC is not needed	16
Data monitoring: interim analysis	#21b	Description of any interim analyses and stopping guidelines, including who will have access to these interim results and make the final decision to terminate the trial	14
Harms	#22	Plans for collecting, assessing, reporting, and managing solicited and spontaneously reported adverse events and other unintended effects of trial interventions or trial	15

For peer review only

		conduct	
Auditing	#23	Frequency and procedures for auditing trial conduct, if any, and whether the process will be independent from investigators and the sponsor	-
**Ethics and dissemination**			
Research ethics approval	#24	Plans for seeking research ethics committee / institutional review board (REC / IRB) approval	2, 18
Protocol amendments	#25	Plans for communicating important protocol modifications (eg, changes to eligibility criteria, outcomes, analyses) to relevant parties (eg, investigators, REC / IRBs, trial participants, trial registries, journals, regulators)	7
Consent or assent	#26a	Who will obtain informed consent or assent from potential trial participants or authorised surrogates, and how (see Item 32)	8
Consent or assent: ancillary studies	#26b	Additional consent provisions for collection and use of participant data and biological specimens in ancillary studies, if applicable	13
Confidentiality	#27	How personal information about potential and enrolled participants will be collected, shared, and maintained in order to protect confidentiality before, during, and after the trial	15
Declaration of	#28	Financial and other competing interests for principal	17

For peer review only

interests		investigators for the overall trial and each study site	
Data access	#29	Statement of who will have access to the final trial dataset, and disclosure of contractual agreements that limit such access for investigators	18
Ancillary and post trial care	#30	Provisions, if any, for ancillary and post-trial care, and for compensation to those who suffer harm from trial participation	-
Dissemination policy: trial results	#31a	Plans for investigators and sponsor to communicate trial results to participants, healthcare professionals, the public, and other relevant groups (eg, via publication, reporting in results databases, or other data sharing arrangements), including any publication restrictions	16
Dissemination policy: authorship	#31b	Authorship eligibility guidelines and any intended use of professional writers	-
Dissemination policy: reproducible research	#31c	Plans, if any, for granting public access to the full protocol, participant-level dataset, and statistical code	-
**Appendices**			
Informed consent materials	#32	Model consent form and other related documentation given to participants and authorised surrogates	-
Biological specimens	#33	Plans for collection, laboratory evaluation, and storage of biological specimens for genetic or molecular analysis in the current trial and for future use in ancillary studies, if applicable	-

For peer review only

None The SPIRIT checklist is distributed under the terms of the Creative Commons Attribution License CC-BY-ND 3.0. This checklist can be completed online using https://www.goodreports.org/, a tool made by the EQUATOR Network in collaboration with Penelope.ai

For peer review only
