# Supplementary material for: Feasibility study of early outpatient review and early cardiac rehabilitation after cardiac surgery: mixed-methods research design—a study protocol
Source: BMJ Open. 2019 Dec 29;9(12):e035787. doi: 10.1136/bmjopen-2019-035787 (PMC6937080; doi:10.1136/bmjopen-2019-035787)
Supplement: Reviewer comments [file bmjopen-2019-035787.reviewer_comments.pdf]

PEER REVIEW HISTORY

BMJ Open publishes all reviews undertaken for accepted manuscripts. Reviewers are asked to complete a checklist review form (**http://bmjopen.bmj.com/site/about/resources/checklist.pdf**) and are provided with free text boxes to elaborate on their assessment. These free text comments are reproduced below.

ARTICLE DETAILS

**TITLE (PROVISIONAL)**	Feasibility Study of Early Outpatient Review and Early Cardiac Rehabilitation After Cardiac Surgery: Mixed Methods Research Design – a study protocol
**AUTHORS**	Ngaage, Dumbor; Mitchell, Natasha; Dean, Alexandra; Hirst, Claire; Akowuah, Enoch; Doherty, Patrick Joseph; Fairhurst, Caroline; Flemming, Kate; Hewitt, Catherine; Hinde, Sebastian; Mitchell, Alex; Nichols, Simon; Watson, Judith

This article was not externally reviewed at BMJ Open. Protocols that have been independently assessed prior to submission to BMJ Open are usually fast-tracked to publication on the grounds that further substantial changes will not be possible. This independent assessment will usually be external, independent review for both a substantial grant award from a non-commercial or government funder and ethics approval.
